# Reduction of NFX1-123 and HPV 16 E6 and E7 Decreased Telomerase and CENP-F in Cervical Cancer Cell Lines

**DOI:** 10.3390/cancers17122044

**Published:** 2025-06-19

**Authors:** Sreenivasulu Chintala, Maura A. Dankoski, Titus K. Maina, Cliff I. Oduor, Kevin M. Quist, Jeffrey A. Bailey, Rachel A. Katzenellenbogen

**Affiliations:** 1 Department of Pediatrics, Indiana University School of Medicine, Indianapolis, IN 46202, USA; srchinta@iu.edu (S.C.); maudanko@iu.edu (M.A.D.); kmquist@iu.edu (K.M.Q.); 2 Department of Pathology and Laboratory Medicine, Brown University, Providence, RI 02903, USA; kipkemboi_maina@brown.edu (T.K.M.); cliff_oduor@brown.edu (C.I.O.); jeffrey_bailey@brown.edu (J.A.B.)

**Keywords:** NFX1-123, human papillomavirus, telomerase activity, hTERT, CENP-F, cervical cancer

## Abstract

Background: Telomerase activity is a cancer hallmark, and hTERT is the rate-limiting catalytic subunit of telomerase. In human papillomavirus type 16 E6 (16E6)-expressing epithelial cells, NFX1-123 augments and is required for full hTERT expression, leading to a growth advantage. However, no studies have investigated the role of NFX1-123 in telomerase activity regulation in HPV-associated cancers. Methods: We knocked out NFX1-123 in CaSki cells (CaSki KO) and performed single-cell RNA sequencing to determine mRNA alterations affected by reduced NFX1-123. Results: In CaSki KO cells, there were three cell clusters based on gene expression, each associated with different enriched biological processes. When pooled and compared with control cells, CaSki KO cells had 1661 decreased and 565 increased mRNAs involving RNA regulation, cell cycle and division, chromatin regulation, and carcinogenesis processes and pathways. CENP-F, a cell cycle and chromosome segregation gene increased in cervical cancers, was among 10 genes with the greatest decrease in mRNA expression in CaSki KO cells. CaSki and SiHa cells with either reduced NFX1-123 or knocked down HPV 16 E6 and E7, demonstrated reduced *hTERT*, *CENP-F*, and telomerase activity, and when both NFX1-123 and HPV 16 E6 and E7 were decreased, *hTERT* and telomerase activity fell further. Finally, *hTERT* and *CENP-F* were increased in cervical cancer primary tumors and in HPV-positive head and neck cancer primary tumors in the TCGA database. Conclusions: These findings highlight the shared role that NFX1-123 has with HPV 16 oncogenes in driving and maintaining RNA, cell cycle, and carcinogenesis pathways, and specifically regulating hTERT, telomerase, and CENP-F.

## Simple Summary

HPV 16 causes cervical cancers and HNSCCs, and NFX1-123, a partner of the HPV 16 E6 oncoprotein, has a high expression in those cancers. Using single cell RNA sequencing, pathway analyses, and TCGA databases, we identified genes that were collaboratively regulated by NFX1-123 and HPV 16 E6. These included *hTERT* and telomerase, as well as *CENP-F*, whose expression was increased in HPV + HNSCC and cervical cancer tumors. These objective studies of HPV + cervical cancer cell lines and HPV-associated cancers permit a better understanding of NFX1-123’s role in molecular, oncogenic alterations and set the foundation for identifying new molecular targets for HPV associated cancers.

## 1. Introduction

The longer splice variant of nuclear transcription factor, X-box binding 1 (NFX1-123) is a cytoplasmic protein that has been recently reported as highly expressed in multiple cancers, including human papillomavirus (HPV)-associated cancers of the cervix and head and neck [1,2,3]. NFX1-123 has been demonstrated as a critical regulator of several genes in normal, diploid human foreskin keratinocytes (HFKs), including human Telomerase Reverse Transcriptase (*hTERT*), the catalytic subunit of the ribonucleoprotein telomerase, through the collaboration of NFX1-123 with cytoplasmic poly(A) binding proteins (PABPCs) and the HPV type 16 E6 oncoprotein (16E6) [4,5,6]. Other collaboratively regulated genes include: *Notch1*, a critical regulator of cell fate [7]; genes involved in keratinocyte differentiation and HPV 16 *L1* gene expression; and genes in the JNK signaling pathway [8]. Additionally, it was shown that NFX1-123, in conjunction with 16E6, subverted immune response genes, creating a milieu permissive for a long-lived HPV infection [9].

The deubiquitinase USP9X, also known as FAF-X, is involved in multiple cancers and is increased by 16E6. USP9X interacts with NFX1-123 and PABPC proteins and stabilizes the NFX1-123 protein itself [10]. When NFX1-123 expression is decreased, pharmacologically or by CRISPR-Cas9 knock out, cervical cancer cell lines have decreased growth, survival, migration, and wound healing, as well as an enhanced cytotoxic efficacy of cisplatin, a widely used chemotherapeutic drug [1]. Beyond human cells, it is important to note that *NFX1* and its homologs are commonly involved in functions associated with gene expression, cancer development, and drug resistance, such as stalled ribosomal protein removal, cellular growth support, drug interaction disruption, and cellular and organismal stress protection [4,11]. Therefore, the roles of *NFX1* in typical cellular functions and in oncogenesis are consistent.

A hallmark of many cancers, including HPV-associated cancers of the cervix and head and neck, is abnormal telomerase activation. Telomerase is normally active in stem cells but not in normal, somatic, diploid cells, such as HFKs; in cancers, telomerase is reactivated and facilitates an escape from cellular senescence and allows oncogenic transformation. The catalytic component of telomerase, hTERT, is considered to be a rate-limiting factor for telomerase activity. Telomerase is proposed as a useful biomarker for the early detection of cervical lesions and a target for anti-cancer therapies in HPV-associated cancers [12], and *hTERT* expression is proportionate to the neoplastic grade of cervical cancers [13]. HPVs have been shown to promote *hTERT* transcription through several mechanisms, including binding to cis-regulatory elements in and removing repressive transcription factors from the *hTERT* promoter, integrating the HPV genome near the *hTERT* locus, and epigenetic upregulation of the *hTERT* promoter [12,14,15,16].

Previously, our laboratory and others have demonstrated the association of two isoforms of nuclear transcription factors, X-box binding 1 (*NFX1*), NFX1-91 and NFX1-123, in *hTERT* regulation with 16E6. 16E6, in conjunction with E6AP, binds to NFX1-91, a transcriptional repressor of the *hTERT* promoter, and degrades NFX1-91 through polyubiquitination to target it for proteasomal degradation. With NFX1-91 removed from the *hTERT* promoter, its expression is derepressed and *hTERT* is transcribed [17,18,19,20,21]. In the cytoplasm, NFX1-123, in cooperation with 16E6 and PABPCs, increases telomerase activity through post-transcriptional stabilization and increased levels of *hTERT* mRNA in keratinocytes [6]. This is one of the mechanisms, leveraging protein partnerships with cellular host proteins, by which HPV-associated cancers can increase hTERT and telomerase activity during oncogenesis.

NFX1-123 high expression has been demonstrated in HPV-associated primary cervical and head and neck cancers and cell lines [1,2,3]. A reduction of NFX1-123 in cervical cancer cell lines leads to decreased hTERT and a slowing of cell growth. Telomerase activity and *hTERT* expression rise with cervical disease severity [13,22,23], and HPV-associated head and neck cancers are more commonly telomerase-positive when compared with non-HPV-associated head and neck cancers [24,25,26]. While it is known that NFX1-123 regulates *hTERT* and telomerase activity in cooperation with 16E6 and PABPCs in HFK models, its exclusive function in gene expression regulation and telomerase activity have not been objectively investigated in cervical cancer models.

Single-cell RNA sequencing (scRNAseq) allows for the identification of cells’ state and type, as well as their clustering based on common gene expression profiles, and it has been widely utilized in heterogeneous tumor tissues to reveal the cellular diversity present in the tumor microenvironment’s cellular architecture. The scRNAseq technique also has advantages in identifying cell clusters in cell culture models [27,28], but this technique has not been well utilized in cancer cell line cultures when an endogenous host gene has been knocked out.

In this study, we knocked out NFX1-123 in CaSki cells (CaSki KO), an HPV 16-positive cervical cancer cell line, to gain a better understanding of its role in heterogenous cell clustering, in gene-expression profiles at the single-cell level, and in telomerase activity in the context of cervical cancer cell lines. We identified three distinct heterogeneous cell clusters at a population level when NFX1-123 was knocked out. When all the clusters were pooled based on NFX1-123 KO versus a control, single-cell gene expression revealed that several genes and pathways associated with cell survival, chromosome stability, infectivity, and host–virus interactions were altered. Among those, *CENP-F*, a kinetochore protein associated with cell cycle regulation and telomerase activity that is highly expressed in breast and cervical cancers, [29,30] showed decreased mRNA and protein expression with reduced NFX1-123. Telomerase activity and *hTERT* mRNA were all significantly decreased in both CaSki and SiHa NFX1-123 KO cells, and this was augmented with siRNA that decreased HPV 16 E6 and E7 expression. In CaSki cells, CENP-F mRNA and protein were decreased with NFX1-123 KO, and siRNA against HPV 16 E6 and E7 further decreased *CENP-F* mRNA. Finally, *hTERT* and *CENP-F* were upregulated in HPV-associated primary cervical and head and neck tumors in the TCGA database compared with normal tissue or non-HPV-associated head and neck tumors.

## 2. Materials and Methods

### 2.1. Cells and Culture Conditions

HPV 16 positive cervical cancer cells SiHa and CaSki were cultured in a DMEM medium supplemented with 10% FBS and 1% penicillin and streptomycin in an incubator at 37 °C with 5% CO_2_. NFX1-123 knock out in SiHa and CaSki cell lines, along with their respective knock-out controls (a non-mammalian gene target), were developed using CRISPR/Cas9 technology as described previously [1]. Cell lines were regularly tested for mycoplasma contamination. To knock down 16E6, the transient transfection of HPV 16 E6E7 siRNA (sense 5′-CUUCGGUUGUGCGUACAAAGC-3′ and antisense 5′-UUUGUACGCACAACCGAAGCG-3′) and a negative control siRNA was performed in CaSki and SiHa NFX1-123 knock out (KO) and controls (CTL) cells using the Lipofectamine 3000 reagent as described by the manufacturer’s instructions (Invitrogen, Carlsbad, CA, USA). For the pharmacological inhibition of NFX1-123, the parental cells of SiHa and CaSki were treated with R428 (1–8 µM) for 72 h as described previously [1].

### 2.2. Single-Cell RNA Library Preparation and Sequencing

NFX1-123 KO CaSki and SiHa cells, as well as a CTL cells, were generated as previously described [1]. Cells were cultured overnight in DMEM at 37 °C, then collected and counted. Approximately 2 × 10^5^ to 5 × 10^5^ cells were cryopreserved in a freezing medium at −80 °C. For single-cell RNA sequencing (scRNAseq), cells were thawed, washed with 1× PBS, and counted using trypan blue to ensure high viability before proceeding with the library preparation.

For single-cell transcriptomic profiling, we employed the Seq-Well platform, a cost-effective, high-throughput strategy for capturing mRNA from thousands of individual cells in parallel [31]. Briefly, following cell counting, the cell suspensions were passed through a 70 µm strainer to remove debris and large aggregates. In total, 10,000–15,000 cells from each sample (CTL and NFX1-123 KO) were loaded onto a functionalized polydimethylsiloxane (PDMS) array preloaded with uniquely barcoded mRNA capture beads (Chemgenes; Wilmington, MA, USA; Macosko-2011-10(V+)). Once the cells settled into the sub-nanoliter wells, the array was sealed with a hydroxylated polycarbonate membrane (10-nm pore size), which facilitated buffer exchange while retaining nucleic acids. Subsequent buffer exchanges permitted cell lysis, mRNA capture by the beads, and bead recovery for reverse transcription. The bead-bound cDNA was then treated with Exonuclease I to remove excess primers, followed by second-strand synthesis and PCR amplification. The resulting cDNA products were purified using SPRIselect beads (Beckman Coulter, Brea, CA, USA) and evaluated on a fragment analyzer (Agilent Technologies, Santa Clara, CA, USA) to confirm fragment sizes of approximately 0.7–2 kb. Libraries were then prepared using the Illumina Nextera XT DNA Library Preparation kit (FC-131-1096) (Illumina, San Diego, CA, USA) with custom primers designed to enrich for 3′ ends of transcripts. Final libraries were purified, quantified, and sequenced on an Illumina NextSeq 550 system (High Output 75-cycle kit) at a target depth of ~20,000 reads per cell. Raw sequencing data and processed UMI count matrices for the CaSki CTL and CaSki NFX1-123 KO experiments have been deposited in the SRA database and are publicly available (BioProject ID: PRJNA1257964).

### 2.3. Single-Cell Transcriptome Analysis

Raw reads were processed using version 2.4.0 of the Drop-seq pipeline, and according to the ‘*Drop-seq tools v2.4.0*’, available at https://github.com/broadinstitute/Drop-seq/releases/tag/v2.4.0 (accessed on 23 April 2025). Demultiplexed FASTQs were aligned to a custom concatenated human–hpv16 reference genome (GRCh38–NC_001526) using STAR aligner, enabling us to capture both human and HPV16 gene expression. Individual reads were tagged with a 12 bp barcode and 9 bp unique molecular identifier (UMI) contained in Read 1 of each sequencing fragment. Following alignment, reads were grouped by the 12 bp cell barcodes and subsequently collapsed by the 9 bp UMI to generate a gene expression count matrix. Gene expression count matrices from CaSki CTL and CaSki KO cell line single-cell experiments were analyzed and visualized in R (v.4.0.3) using the Seurat package (v.4.2). Cells with less than 300 genes and UMIs detected in less than ten cells were excluded from further analysis. The library sizes for each group in the study were internally normalized to 10,000 transcripts, log transformed, and regressed on the number of UMIs per cell before dimensionality reduction and clustering. Datasets were integrated by utilizing the canonical correlation analysis (‘CCA’) in Seurat to identify shared sources of variations between each group to learn the cell lines’ gene expression changes associated with the knock out of the NFX1-123 gene. Next, the read data were scaled and analyzed via the principal component analysis (PCA). The top principal components, PCs (*n* = 20), were dimensionally reduced via uniform manifold approximation (UMAP) [32] and unsupervised clustering was performed at several resolutions to identify the cell clusters within the cell lines and analyze differential gene expression between the two groups. A *p*-value cutoff of 0.05 was used to determine significance. For differential gene expressions (DGEs) between the two groups, a Bonferroni correction was applied to generate the adjusted *p*-values. A DGE analysis was performed using the MAST package v1.28.0 (https://bioconductor.org/packages/MAST.html, accessed on 23 April 2025).

### 2.4. Gene Ontology Analysis of Differentially Expressed Genes Across Cell Clusters

To elucidate the biological functions and pathways associated with the differentially expressed genes (DEGs) observed in the NFX1-123 KO CaSki cell line compared with the CaSki CTL cells, a gene ontology enrichment analysis was performed using the gprofiler suite [33] in the three cell clusters (0, 1, and 2). Briefly, the list of the DEGs between the CaSki CTL and CaSki KO was uploaded to the g:GOST functional enrichment analysis module (https://biit.cs.ut.ee/gprofiler, accessed on 23 April 2025) within gProfiler. GO enrichment was evaluated across three principal GO domains, biological process, molecular function, and cellular component. The gprofiler’s default settings were relied upon, which applied the Benjamini–Hochberg correction to control false discoveries. GO terms with an adjusted *p*-value (false discovery rate, FDR) below 0.05 were considered statistically significant. The charts generated in each cluster include: Source listing the GO term Molecular Function (GO:MF), Biological Process (GO:BP), and Cellular Component (GO:CC); Term ID with unique GO ID number; Term Name (enriched terms associated with the gene list) listing the specific term associated with the source; and Padj(query_1) − Adjusted *p*-value (false discovery rate, FDR). Specific molecular functions and biological processes enriched in the Cluster 1 cell population were noted with a star, based on their relative association with the functions of NFX1-123, cancer and oncogenesis, and infection-associated cellular pathway changes.

### 2.5. RNA Isolation and Gene Expression Profiling by PCR

Total RNA was isolated from cell lines using the RNeasy Mini Kit (Qiagen, Germantown, MD, USA). Briefly, the cells were cultured in 6-well plate dishes, and cell pellets were collected. Cell pellets were lysed with Buffer RLT, total RNA isolation was performed per the manufacturer’s instructions, and the RNA was eluted with 30 µL of RNase-free water. The total RNA quantity and purity were verified using the Nanodrop Spectrophotometer. All experiments were repeated with same number of cells and conditions.

Total RNA was used to prepare cDNA using SuperScript IV VILO with the ezDNase enzyme kit (Invitrogen, Carlsbad, CA, USA). Briefly, 10 µL of gDNA digestion reaction mix, for RT and no-RT reactions, in an RNase-free tube were placed on ice with a 10× ezDNase Buffer, ezDNase enzyme, and total RNA, with a final volume of 10 µL with nuclease-free water. This was gently mixed and incubated at 37 °C for 2 min. Then, 4 µL of Superscript IV VILO Master mix, or Superscript IV VILO No RT Control, and 6 µL of nuclease-free water were added to the tube containing 10 µL of the reaction mix. The mixture was incubated at 25 °C for 10 min, 50 °C for 10 min, and 85 °C for 5 min. cDNA was used to quantify gene expression using qPCR using the PowerUp SYBR Green Master Mix kit (Applied Biosystem, Carlsbad, CA, USA). A list of primers used for the individual qPCRs are below. 36B4 was used as internal loading qPCR control standard, as previously published [3].

- INHBA-F: 5′-AAGTCGGGGAGAACGGGTATG-3′
- INHBA-R: 5′-TCTTCCTGGCTGTTCCTGAC-3′
- NEAT1-F: 5′-CCAGTTTTCCGAGAACCAAA-3′
- NEAT1-R: 5′-ATGCTGATCTGCTGCGTATG-3′
- RBM25-F: 5′-TGTCTTTTCCACCTCATTTGAATCG-3′
- RBM25 R: 5′-ATTGGTACAGGAATCATTGGGGT-3′
- PRRC2C-F: 5′-CCATCAGTAGCAAAAGTTCCC-3′
- PRRC2C-R: 5′-CTTCGCTCTTCCTCTTCACG-3′
- NEFM-F: 5′-TCAACGTCAAGATGGCTCTG-3′
- NEFM-R: 5′-GAGCTTCCACCTTGGGTTTC-3′
- DYNC1H1-F: 5′-GCCACCGTCAGTTTTGACAC-3′
- DYNC1H1-R: 5′-AAATTGCCTCCACCAAACGC-3′
- MKI67-F: 5′-CGTCCCAGTGGAAGAGTTGT-3′
- MKI67-R: 5′-CGACCCCGCTCCTTTTGATA-3′
- FTL-F: 5′-CAGCCTGGTCAATTTGTACCT-3′
- FLT-R: 5′-GCCAATTCG CGGAAGAAGTG-3′
- SAT1-F: 5′-GAGGCTTTGGCATAGGATCA-3′
- SAT1-R: 5′-TCCAACCCTCTTCACTGGAC-3′
- CENPF-F: 5′-CTCTCCCGTCAACAGCGTTC-3′,
- CENPF-R: 5′-GTTGTGCATATTCTTGGCTTGC-3′
- hTERT-F: 5′-CGAGCTGCTCAGGTCTTTCTTTTATG-3′
- hTERT-R: 5′-CCACGACGTAGTCCATGTTCACAATC-3′
- 36B4-F: 5′-TGCCAGTGTCTGTCTGCAGA-3′
- 36B4-R: 5′-ACAAAGGCAGATGGATCAGC-3′

A total reaction volume of 10 µL was used in technical triplicate samples for each qPCR, and a no RT template control reaction was performed to detect PCR contamination. The target gene expression was compared with 36B4, using the comparative ΔΔCt values. The mRNA expression fold changes between CTL and KO were analyzed for statistical significance using an unpaired *t*-test using GraphPad Prism version 10.0.0 for Windows, GraphPad Software, Boston, MA, USA, www.graphpad.com. The significance was noted using *p*-values (minimum of <0.05).

### 2.6. Telomerase Activity Assay

Telomerase activity was evaluated using the TRAPeze Telomerase Detection kit protocol, non-radioactive detection option (Millipore, Burlington, MA, USA). CaSki and SiHa NFX1-123 KO or CTL, HPV 16 E6 and E7 knock down or control, and R428 treated or vehicle control were used to the evaluate telomerase activity, with telomerase-positive cell pellets provided by the kit as a positive control. Cell pellets were lysed in 200 µL of 1× CHAPS lysis buffer containing 200 units/mL of RNase inhibitor. The cell suspension was incubated on ice for 30 min and spun at 12,000× *g* for 20 min at 4 °C. The supernatant was then collected. Protein concentrations of the cell extracts were determined using the Bio-Rad protein quantification reagent (Bio-Rad, Hercules, CA, USA). For the TRAP assay itself, a master mix was prepared per the kit instructions. The TSK2 PCR amplification internal control (IC), which produces a 36-bp band, was added to each reaction. In total, 48 µL of reaction master mix were aliquoted into RNase-free PCR tubes. In total, 2 µL of each cell extract, containing 50 ng/µL protein, were added to the reaction mix. Controls included: telomerase quantitative control template TSR8 (2 µL) for the standardization of telomerase activity; a primer dimer/PCR contamination negative control (2 µL 1 × CHAPS lysis buffer or 2 µL of molecular grade water); and a heat-inactivated cell extract (2 µL) negative control. Tubes were placed in a thermocycler block to perform a PCR amplification: 30 °C for 30 min; 95 °C for 2 min; then 30 cycles of 94 °C for 15 s, 59 °C for 30 s, and 72 °C for 1 min.

PCR-amplified products were separated on a 20% polyacrylamide (PAGE) gel. The gel was prepared using an acrylamide/bis-acrylamide 40% solution 19:1 (Fisher Chemical, Fair Lawn, NJ, USA), and the Variable Comb Vertical (VCV) System Electrophoresis tank (IBI Scientific, Dubuque, IA, USA) was used to run the gel. In total, 60 mL of resolving gel were prepared with 30 mL of 40% solution 19:1 acrylamide/bis-acrylamide, 15 mL of 1.5 M Tris HCl pH 8.8 buffer, 0.5 mL of 10% ammonium per sulfate, 0.1 mL of TEMED, and 14.4 mL of distilled water. It was poured into the assembled glass plates and allowed to polymerize. A 12.5 mL stacking gel (4%) was prepared with 1.25 mL of acrylamide/bis-acrylamide 40% solution 19:1, 3.15 mL of 0.5 M Tris HCl pH 6.8 buffer, 0.125 mL of 10% ammonium per sulfate, 0.05 mL of TEMED, and 12.5 mL of distilled water. It was poured on the top of the resolving gel and a comb was inserted. After polymerization, the gel was placed in the vertical gel tank, and a TBE buffer was poured in the upper and lower tanks. In total, 18 µL of PCR amplified product were mixed with 3 µL of 6× loading dye and loaded into the wells. In total, 4 µL of low-range DNA markers were loaded for the size determination of PCR products. Samples were run at 300 volts for 4–5 h. The gene was stained with ethidium bromide (Fisher Scientific, Fair Lane, NJ, USA) or SYBR Safe (Invitrogen, Carlsbad, CA, USA) and images were collected using the ChemiDoc Imager. An ImageJ (Image J 1.54b, Java 1.8.0_322 (64-bit)) analysis measured the density of telomerase activity using PCR product bands and the percent telomerase activity was calculated relative to the control cells as 100% activity, as previously published [5].

### 2.7. Western Blot

Whole cell protein extracts were collected using the Triton X-100 protein lysis buffer (5mMTris HCl pH 7.5, 15 mM NaCl, and 1% Triton X-100). Protein quantification was performed using the Bio-Rad reagent (Hercules, CA, USA). In total, 20–40 µg of protein were electrophoresed on a 4–20% gradient SDS gel and transferred using Tran-Blot Turbo Mini 0.2 µm PVDF Transfer Packs in the Trans-Blot Turbo Transfer System (Bio-Rad, Hercules, CA, USA). PVDF membranes were blocked for 1 h using a blocking agent (Amersham ECL Prime, Cytiva, Marlborough, MA, USA), and primary antibodies NFX1-123 (Novus Biologicals, anti-rabbit, NBP1-49933, 1:1000 dilution), p53 (Millipore Sigma, Burlington, MA, USA, anti-mouse, OP43, 1:500 dilution), CENP-F (Abcam, Waltham, MA, USA, anti-rabbit, ab5, 1:250 dilution) and GAPDH (Abcam, anti-mouse, ab8245 1:50,000 dilution) were added, incubating at 4 °C overnight. PVDF membranes were then washed for 30 min with PBS-T, and HRP-conjugated anti-rabbit (Cell Signaling, 7074S, Danvers, MA, USA, 1:5000 dilution) or anti-mouse (Cell Signaling, 7076S, Danvers, MA, USA, 1:10,000 dilution) secondary antibodies were added to the PVDF membranes, incubating for 1 h at room temperature. PVDF membranes were then washed for 30 min with PBS-T, developed with an ECL Prime detection reagent, and images were collected using the ChemiDoc Imaging System. (Bio-Rad, Hercules, CA, USA)

### 2.8. TCGA Database Gene Expression Analysis in HPV-Associated and Non-HPV-Associated Cancers

TCGA data for hTERT and CENP-F mRNA expression in HPV-associated cancers of the cervix and head and neck were downloaded from the TCGA Splicing Variants Database (TSVdb). HPV-associated and non-associated primary tumors from head and neck cancers were identified and compared with each other and with normal solid tissue. Cervical cancer primary tumors were compared with normal solid tissue. A graphical presentation of hTERT and CENP-F mRNA expression was created using GraphPad Prism version 10.4.0 (621). A statistical analysis was performed using the Mann–Whitney test to determine the *p*-values between each group.

### 2.9. DAVID Bioinformatic Analysis of Differentially Expressed Genes in CaSki Knock Out and Control Cells

Downregulated genes in CaSki KO cells (top 12%, *n* = 210) were used to perform a DAVID Bioinformatic analysis. The DAVID Knowledgebase (v2023q4) was used to identify biological processes, molecular functions, and pathways associated with the downregulated genes as described [34,35]. The charts generated included: Category (resource/original database where the terms orient); Term (enriched terms associated with the gene list); RT (related term search); Genes Count (number of genes involved in the term); % (percent of genes involved out of total genes included); *p*-Value (EASE Score, the modified Fisher Exact *p*-value); and Benjamini (adjusted *p*-value using linear step-up methods). Specific enriched biological processes, molecular functions, and pathways were noted based on their relative association with the functions of NFX1-123, cancer and oncogenesis, and infection-associated cellular pathway changes.

## 3. Results

### 3.1. Three Cell Clusters Identified Using Single-Cell RNA Sequencing of NFX1-123 Knock Out CaSki Cells

**Figure 1:**
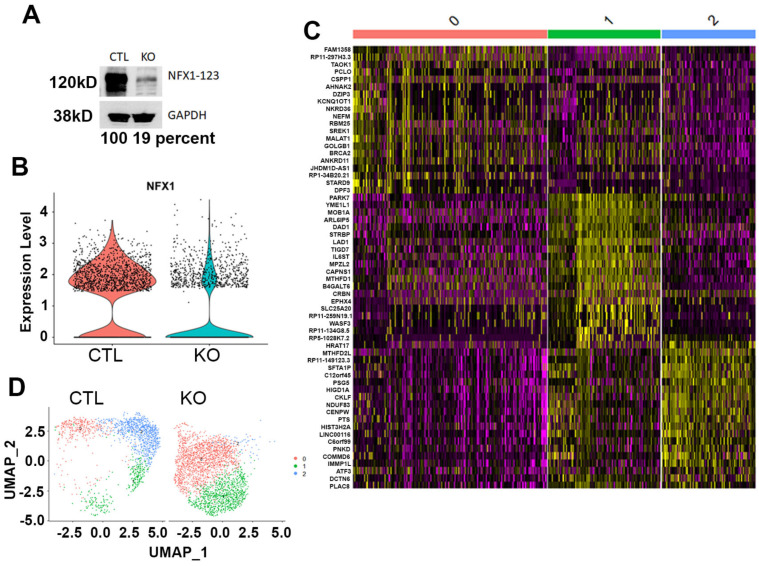
NFX1-123 knock out in CaSki cells identified the three cell clusters based on gene expression differences. (**A**) Western blot analysis showing a significant decrease in NFX1-123 protein to 19% in CaSki KO cells. GAPDH was used as internal control. (**B**) Single-cell sequencing analysis showing the NFX1 mRNA expression in NFX1-123 KO and control cells. (**C**) Heatmap showing scaled expression (Z-score) of the 20 most upregulated genes per Seurat cluster (rows) across all CaSki single cells (columns). Columns are grouped and annotated by cluster identity (color bar above), and genes are grouped by similarity in their expression profiles. The color scale indicates low (purple) to high (yellow) relative expression, highlighting the transcriptional signatures that define each cell cluster. (**D**) UMAP projection plot of CaSki cells (control (CTL) and NFX1-123 knock out (KO)), colored by Seurat-defined clusters (0, 1, and 2). Each point represents one cell in the two-dimensional UMAP space computed from the first 20 principal components of the normalized gene expression matrix to define how global transcriptional profiles separated the cells according to their treatment. The original Western blot figures can be found in Supplementary File S1.

Compared with CRISPR/Cas9 control (CTL) CaSki cells, CRISPR/Cas9 knock out (KO) of NFX1-123 had significantly decreased protein, using a western blot (Figure 1A), and RNA expression, using an scRNAseq analysis (Figure 1B). Once confirmed, scRNAseq data from CaSki KO and CaSki CTL cells were merged and analyzed, and three distinct cell clusters were identified (0, 1, and 2) using a gene expression heatmap (Figure 1C) and UMAP (Figure 1D). When separated, CaSki KO cells were enriched in Clusters 0 and 1 when compared with CaSki CTL cells (Figure 1D). This shift in cluster representation, even without a full knock out of NFX1-123 expression, highlighted the impact of NFX1-123 on the gene expression patterns of CaSki cells. The heatmap further highlights these transcriptional differences, indicating that NFX1-123 depletion influenced cellular behavior and contributed to the distinct clustering observed in the scRNAseq data.

### 3.2. Biological Processes Enriched in NFX1-123 KO Cell Clusters

**Figure 2:**
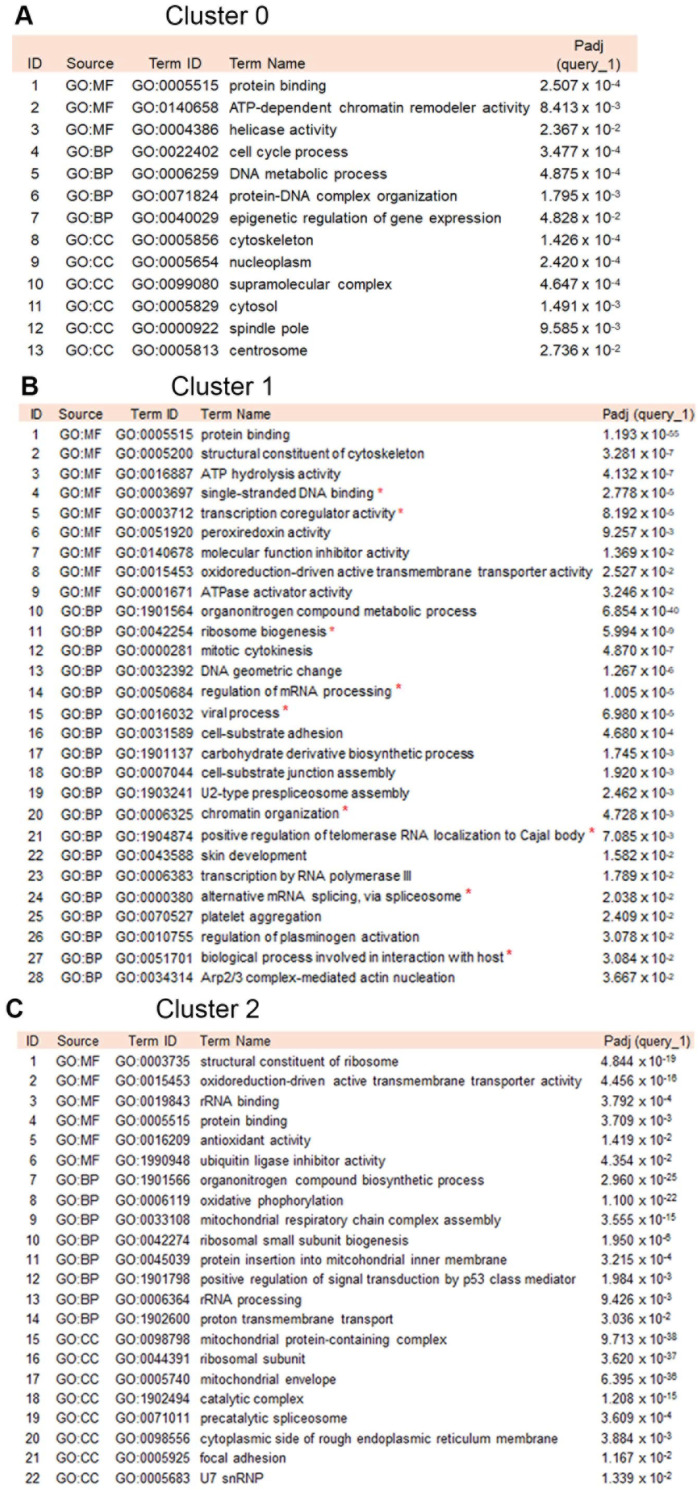
Gene Ontology (GO) analysis of biological processes enriched in three clusters of cells. (**A**) Cluster 0, (**B**) Cluster 1, and (**C**) Cluster 2 cells showed enrichment of biological processes, with the most occurring in Cluster 1. Relevant term names in Cluster 1 associated with NFX1-123 are highlighted with a red star symbol.

To examine the biology and functions of the differentially expressed genes between control and CaSki KO cells in clusters 0, 1 and 2, a gene ontology (GO) enrichment analysis for molecular functions (GO:MF), biological processes (GO:BP), and cellular components (GO:CC) was performed (Figure 2). The comparison of BPs and MFs among the three clusters, and even more so for Cluster 1 that was primarily populated with CaSki KO cells, showed an enrichment of several known HPV- and NFX1-123-associated biological processes and molecular functions. These included ribosome biogenesis, viral process, regulation of mRNA processing, interaction with the host, chromatin organization, positive regulation of telomerase RNA localization, and alternative mRNA splicing via spliceosome (marked with red stars in Figure 2B). This data implied that the reduction of NFX1-123 in CaSki cells led to significant changes in key pathways that regulate gene expression, RNA, telomerase, and viral infections, even without direct changes to the partner of NFX1-123: the 16E6 gene expression itself.

### 3.3. Differentially Expressed Genes in NFX1-123 KO CaSki Cells

**Figure 3:**
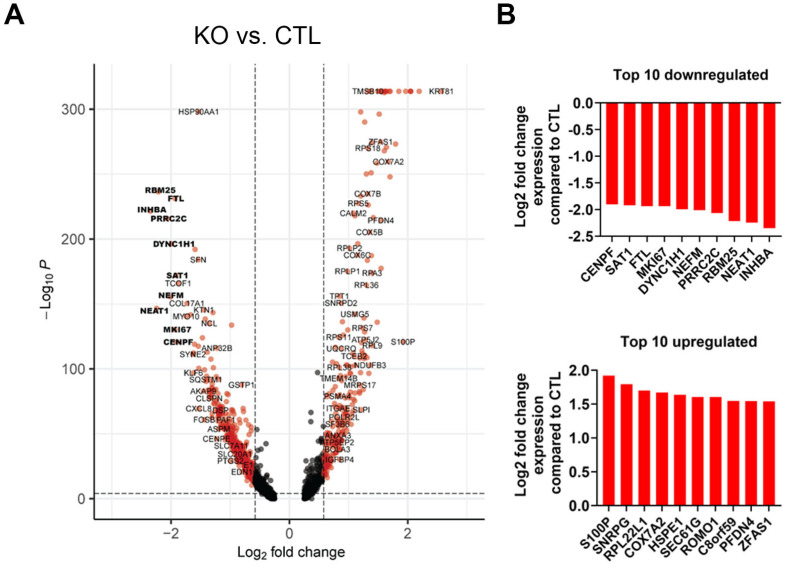
NFX1-123 knockdown resulted in the differential expression of genes in CaSki KO cells at the single cell level. (**A**) Differential gene expression profile of CaSki cells following NFX1-123 knock out. Volcano plot illustrating the results of a single-cell differential expression analysis between KO and CTL CaSki cells. Each point represents a gene; the *x*-axis shows log_2_ fold change (KO vs. CTL) and the *y*-axis depicts the –log of the BH-adjusted *p* value (MAST test, Benjamini–Hochberg correction). Vertical dashed lines mark ±0.58 log_2_ fold change (≈1.5 fold), and the horizontal dashed line indicates the significance threshold of adjusted *p*-value < 0.01. Genes that met both thresholds are colored red. Notably, several genes involved in proliferation, RNA processing, and cytoskeletal organization (*INHBA*, *NEAT1*, *RBM25*, *PRRC2C*, *NEFM*, *DYNC1H1*, *MKI67*, *FTL*, *SAT1*, and *CENPF*) are significantly downregulated in NFX1-123 KO cells (highlighted in bold). (**B**) Ten most downregulated (upper) and upregulated (lower) genes in NFX1-123 KO CaSki cells compared with CTL.

**Figure 4:**
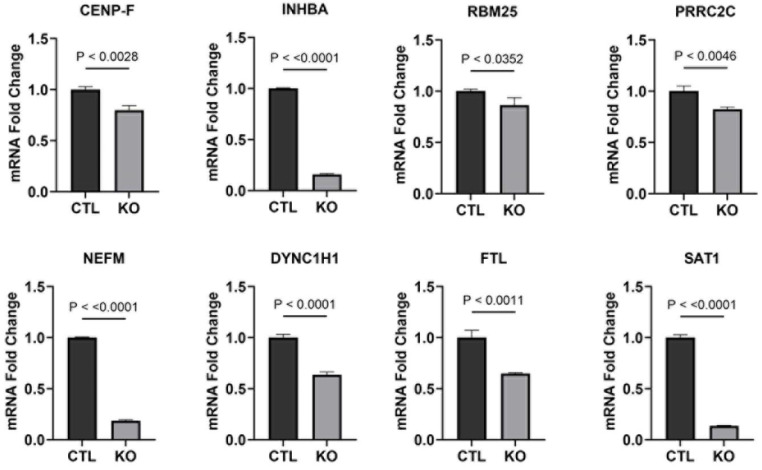
Confirmation of downregulated genes using qPCR in NFX1-123 KO CaSki cells. Expression of mRNA of downregulated genes was determined using qPCR. Fold change in the expression, with CTL set as 1.0, was shown in CTL and KO CaSki cells. Statistical significance is shown as *p* values.

To evaluate the effect of NFX1-123 on gene expression, differentially expressed genes (DEGs) identified using scRNAseq in pooled CaSki KO cells compared with CaSki CTL cells were evaluated. As demonstrated by a volcano plot, 2228 genes had significantly changed expression in CaSki KO cells (1661 decreased with a log2 fold change of −0.25 to −2.3, and 565 increased with a log2 fold change of 0.25 to 2.6) (Figure 3A). Ten genes with the greatest decrease or increase in expression using scRNAseq, and their specific average fold change, are shown (Figure 3B, decreased expression top panel, increased expression lower panel). Quantitative PCR confirmed eight out of ten (80%) downregulated genes using scRNAseq that were reduced in NFX1-123 KO CaSki cells (Figure 4). These results validated the general expression shifts identified using scRNAseq. The differentially expressed genes identified were involved in cellular secretion, cell development and maturation, RNA binding, intracellular motility, protein sorting, iron homeostasis, polyamine metabolism, chromosome segregation, and telomerase activity—processes that closely align with the known functions of NFX1-123 (Supplemental Table S1).

### 3.4. Gene Ontology Analysis of Decreased Genes in NFX1-123 KO CaSki Cells

**Figure 5:**
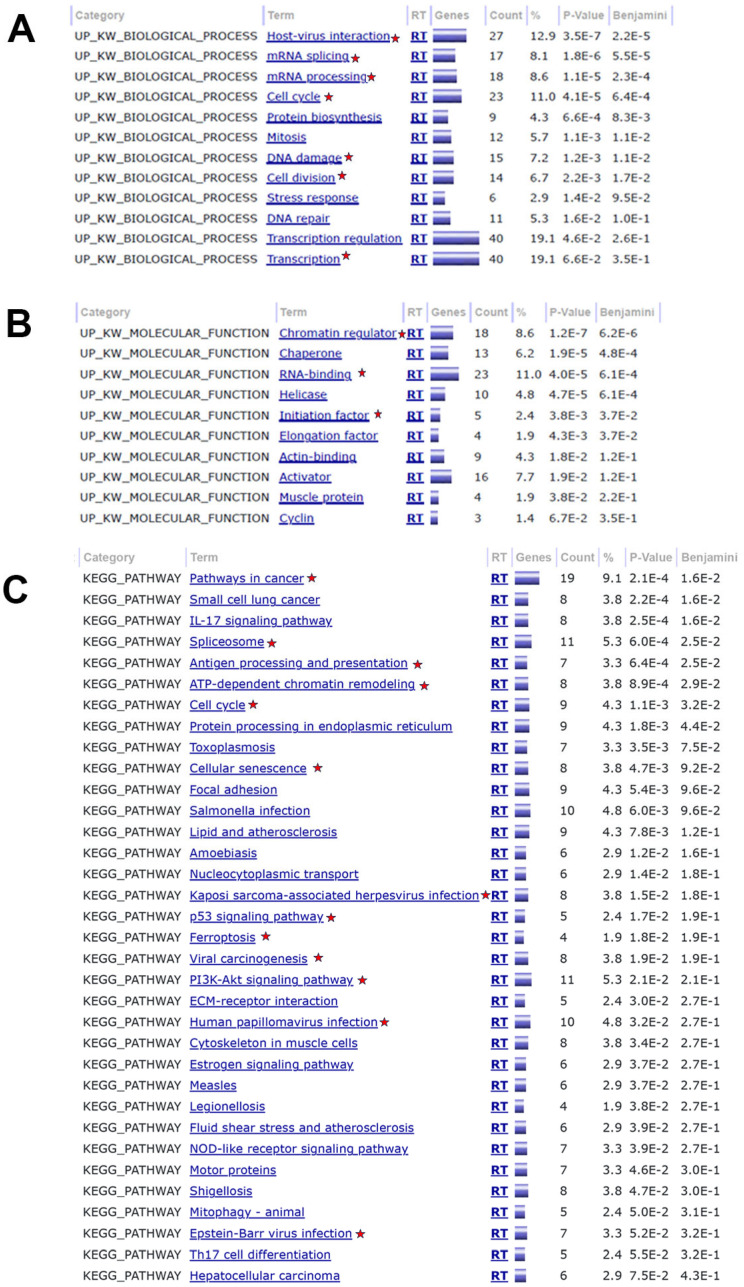
DAVID bioinformatics analysis of top 210 downregulated genes associated with biological processes, molecular functions, and pathways. (**A**–**C**) Critical, relevant terms are noted with a red star symbol (pentagram). (**A**) Enriched biological processes associated with NFX1-123 downregulated genes. (**B**) Enriched molecular functions with downregulated genes. (**C**) Pathway analysis showed 12 critical pathways enriched with the downregulated genes.

After identifying and validating the genes that were most downregulated in NFX1-123 KO relative to CTL CaSki cells, genes with a decreased expression (log_2_ fold change −0.90 to −2.35, *n* = 210, 12% total) were analyzed using DAVID Bioinformatics. Significant alterations in biological processes were seen, such as host–virus interactions, mRNA processing, splicing, cell cycle, cell division, DNA damage, and transcription (Figure 5A, starred). The downregulated genes also suggested a shift in key molecular functions, including RNA binding, chromatin regulation, activator, chaperone, helicase, and actin binding changes that aligned with the known roles of NFX1-123, its homologs, and tumor-associated viruses [5,6,36] (Figure 5B, starred). Multiple KEGG pathways in cancer, HPV infection, Kaposi Sarcoma-associated herpesvirus infection, viral carcinogenesis, Epstein–Barr virus infection, cell cycle, ATP-dependent chromatin remodeling, spliceosome, cellular senescence, ferroptosis, antigen processing and presentation, and the p53 signaling pathway were affected in CaSki cells with NFX1-123 knock out (Figure 5C, starred). It is important to note that, in NFX1-123 KO CaSki cells, HPV 16 and its gene expression were not directly affected, only NFX1-123; however, the downstream effects of reduced NFX1-123 expression led to cellular gene expression functions, processes, and pathway changes that are associated with HPV and HPV-associated cancers.

### 3.5. NFX1-123 Knock Out Decreased Telomerase Activity, hTERT, and CENP-F

**Figure 6:**
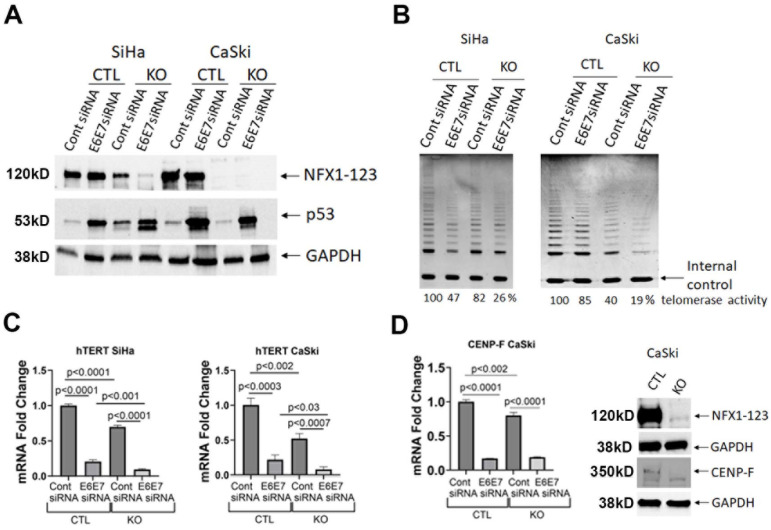
NFX1-123 knock out deceased telomerase activity, hTERT, and CENP-F in cervical cancer cells. (**A**) NFX1-123 protein expression was downregulated in NFX1-123 KO SiHa and Caski cells compared with CTL. HPV 16 E6 and E7 decrease by E6E7 siRNA compared with Cont SiRNA was confirmed by p53 upregulation. GAPDH served as an internal control. (**B**) Telomerase activity in SiHa and CaSki cells was reduced with NFX1-123 KO and with E6E7 knock down. The percent telomerase activity was noted for each cell line as compared with CTL/Cont siRNA set at 100% activity. (**C**) Quantitative PCR analysis of *hTERT* mRNA in CTL and NFX1-123 KO SiHa and CaSki cells. mRNA expression fold changes were calculated and normalized to the internal control. The studies were conducted in triplicate, and error bars represent the standard error of mean (SEM). Statistical analysis of a standard *t*-test was performed, and significant differences are noted with *p* values. (**D**) *CENP-F* mRNA and protein were decreased with NFX1-123 KO in CaSki cells. GAPDH served as an internal control. The original Western blot figures can be found in Supplementary File S1.

Telomerase activity is universally seen in cervical cancers, and its activation permits cells to avoid cellular senescence signals due to the Hayflick limit [37]. Since cellular senescence, pathways in cancer, viral carcinogenesis, and HPV infection were all affected in NFX1-123 KO CaSki cells, and NFX1-123 and 16E6 have been shown to collaboratively regulate hTERT and telomerase in human foreskin keratinocytes [5,6,36], we aimed to determine the role of NFX1-123, singularly or in concert with 16E6, in hTERT and telomerase in HPV 16+ cervical cancer cell lines and cervical cancers. Additionally, CENP-F RNA expression was dramatically reduced in NFX1-123 KO CaSki cells (Figure 4 and Figure 6D). Recent studies have shown CENP-F to be highly expressed in cervical cancer tissues and cells [38], and CENP-F expression correlates with high telomerase activity in breast cancer [29]. With that, NFX1-123 KO and knock down of HPV 16 E6 and E7 using siRNA (E6E7), or their controls, was generated singularly or dually in both CaSki and SiHa cell lines to quantify the effect that their reduction had on telomerase activity, *hTERT* mRNA expression, and CENP-F.

The NFX1-123 protein was decreased in SiHa KO cells and completely reduced in CaSki KO cells, and the p53 protein rebounded with siRNA E6E7 knock down (Figure 6A). Telomerase activity (Figure 6B) was decreased by nearly 20% in SiHa KO cells that retained some NFX1-123 expression but was reduced by 60% in CaSki KO cells with no detectable NFX1-123 expression. Itself, siRNA E6E7 knock down lowered telomerase activity by more than 50% in SiHa cells and 15% in CaSki cells. With both NFX1-123 KO and siRNA E6E7, SiHa and CaSki cells had telomerase activity that decreased by nearly 75% and more than 80%, respectively (Figure 6B). Broadly, reduced *hTERT* mRNA correlated with decreased telomerase activity in both cell lines either with NFX1-123 KO or siRNA E6E7 compared with controls; however, an augmented decrease in hTERT was seen with reductions in both NFX1-123 and 16E6 (Figure 6C). The CENP-F protein was nearly absent in NFX1-123 KO CaSki cells (Figure 6D), associating telomerase activity, *hTERT* mRNA, and CENP-F protein expression with NFX1-123 and 16E6 in cervical cancer cell lines.

### 3.6. Pharmacological Inhibition of NFX1-123 Decreased Telomerase Activity, hTERT, and CENP-F

**Figure 7:**
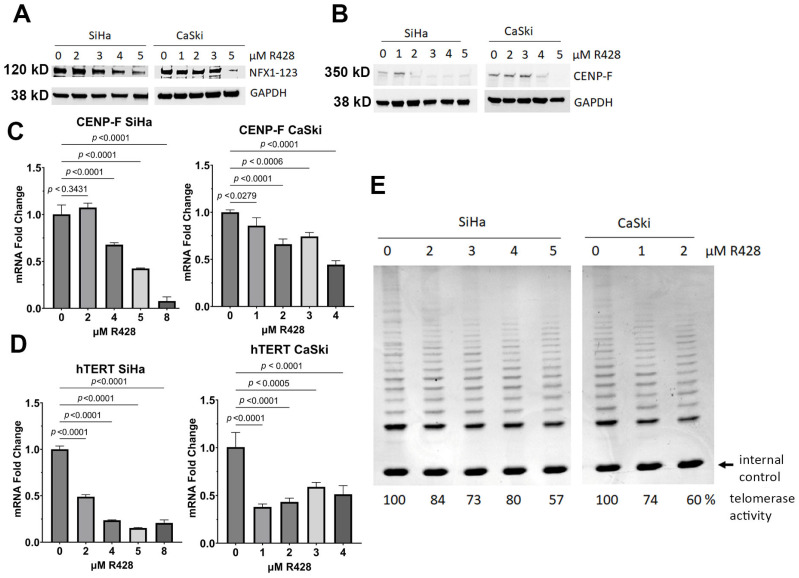
Pharmacological inhibition of NFX1-123 correlated with dose-dependent, reduced CENP-F, hTERT, telomerase activity in SiHa and CaSki cells. (**A**) NFX1-123 had R428 dose-dependent decreases in cells. GAPDH served as an internal control. (**B**) CENP-F protein had R428 dose-dependent decreases in cells. GAPDH served as an internal control. (**C**) *CENP-F* mRNA had a R428 dose dependent-decrease in cells. Expression normalized to the internal control. (**D**) *hTERT* mRNA had a R428 dose-dependent decrease in cells. Expression normalized to the internal control. The studies were conducted in triplicate, and error bars represent the standard error of the mean (SEM). (**E**) Telomerase activity had a R428 dose-dependent decrease in cells. The original Western blot figures can be found in Supplementary File S1.

In complement to the KO studies of NFX1-123 in CaSki and SiHa cells, we aimed to evaluate whether the pharmacological reduction of NFX1-123 would also lower telomerase activity, *hTERT* mRNA, and *CENP-F* mRNA and protein expression. We have identified R428 as a drug compound that decreases NFX1-123 protein levels [1], and cell lines treated with R428 had decreased NFX1-123 protein [1]. In SiHa and CaSki cells treated with R428, the NFX1-123 protein was reduced (Figure 7A), and telomerase activity was decreased (Figure 7E). Like in CaSki and SiHa KO cell lines, *hTERT* mRNA and *CENP-F* mRNA and protein were reduced in cells treated with R428. (Figure 7B–D). These results demonstrated that pharmacological inhibition, in addition to targeted genetic knock out, of NFX1-123 led to decreased telomerase activity, hTERT, and CENP-F.

### 3.7. High-Risk HPV Increased hTERT and CENP-F mRNA in Primary Tumors of HPV-Associated Cancers

**Figure 8:**
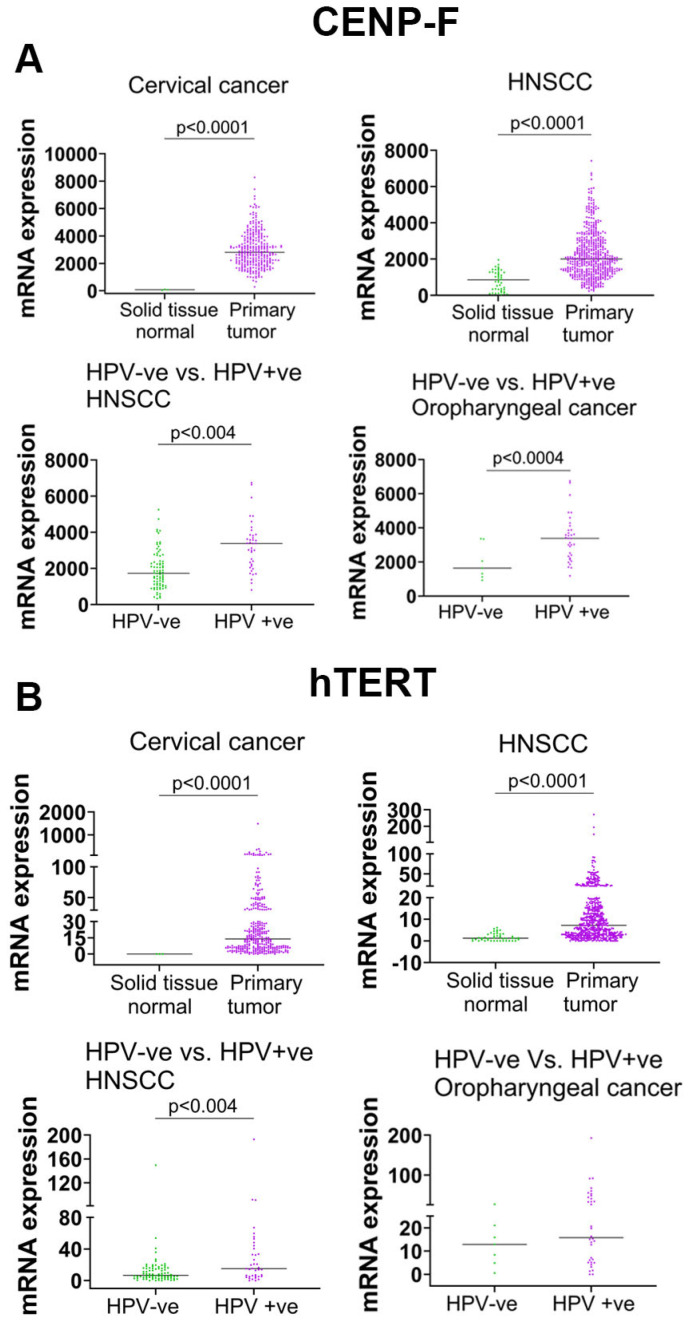
*CENP-F* and *hTERT* were significantly upregulated in HPV-positive primary tumors. (**A**) *CENP-F* mRNA was significantly increased in cervical cancer (*n* = 304) and HNSCC (*n* = 520) primary tumors compared with normal solid tissue (cervical *n* = 3; HNSCC *n* = 44). HPV-positive HNSCCs (*n* = 39) and oropharyngeal cancers (*n* = 34) expressed significantly higher levels of CENP-F compared with HPV-negative HNSCC (*n* = 80) and oropharyngeal cancers (*n* = 7). (**B**) *hTERT* mRNA was significantly increased in cervical cancer (*n* = 304) and HNSCC (*n* = 520) primary tumors compared with normal solid tissue (cervical *n* = 3; HNSCC *n* = 44). HPV-positive HNSCCs (*n* = 39) expressed significantly higher levels of hTERT compared with HPV-negative HNSCCs (*n* = 80). There was an increased trend of *hTERT* mRNA in HPV-positive oropharyngeal cancers (*n* = 34) compared with HPV-negative oropharyngeal cancers (*n* = 7).

While telomerase, hTERT, and CENP-F are known to be universally detected in cervical cancers, the expression of *hTERT* and *CENP-F* in HPV-associated cancers more broadly has not been quantified. Using the TCGA database, *CENP-F* and *hTERT* mRNA expression in cervical cancers revealed a significant upregulation of *CENP-F* and *hTERT* mRNA in the primary tumors of cervical cancers when compared with normal cervical tissues (Figure 8A,B). Primary head and neck squamous cell carcinoma (HNSCC) tumors also expressed significantly higher levels of *CENP-F* and *hTERT* transcripts compared with normal solid tissue. When parsing the role of HPV in HNSCCs and the expression of CENP-F hTERT, HPV-positive HNSCC tumors had higher *CENP-F* and *hTERT* expression than HPV-negative HNSCC tumors. These findings indicate that HPV is associated with increased hTERT expression in cancers across anatomic sites, and NFX1-123 in vivo expression levels correlate with a greater expression of CENP-F and hTERT in HNSCCs (Figure 8) [3].

## 4. Discussion

NFX1-123, an RNA binding protein and known host protein partner of 16E6, has been shown to be involved in the regulation of several cellular pathways, including cellular growth, survival, differentiation, and immune regulation [4]. NFX1-123 is highly expressed in HPV-associated cancers of the cervix [2] and the head and neck [3]. In cervical cancer cell lines, pharmacological inhibition and reduction of NFX1-123 result in decreased cancer cell growth, survival, migration, and invasion, as well as enhanced cisplatin cytotoxic effects [1]. These studies and findings indicate the important role that NFX1-123 may play in cancer cell growth and survival, especially in HPV-associated cancers. However, the precise function of NFX1-123 in cellular gene expression and regulation has not been objectively investigated in depth. In this study, we sought to better understand the role of NFX1-123 using an scRNAseq analysis of cervical cancer cell lines. From our scRNAseq data, we identified three distinct clusters of CaSki cells (Figure 1). Clusters 0 and 1 were predominantly enriched by cells where NFX1-123 was knocked out by CRISPR/Cas9 and had variable reductions in *NFX1-123* mRNA expression (Figure 1B). The emergence of distinct subpopulations of cells in a two-dimensionally grown cancer cell line is consistent with previous observations of heterogeneity in cultured tumor cells [28]. Notably, Cluster 1 showed enrichment in processes such as chromatin organization, telomerase RNA localization, mRNA processing, ribosome biogenesis, viral processes, and viral–host interactions (Figure 2B). These findings suggest that NFX1-123 KO contributes to differential molecular architectures, giving rise to subpopulations with unique functional profiles, as has been demonstrated in other single-cell analyses [27,28].

In our scRNAseq analysis, we identified 2208 genes that were differentially expressed between NFX1-123 KO and control CaSki cells, with 75% of these genes displaying reduced expression. This indicates that NFX1-123 supports the elevated expression of a broad set of genes. Of the ten most downregulated genes (using scRNAseq) in NFX1-123 KO CaSki cells, eight of ten were confirmed to be significantly reduced using qPCR (Figure 4). These validated genes included *RBM25* and *PRRC2C* (RNA binding proteins), *DYNC1H1* (microtubule activation protein), *FLT* (iron regulation), *CENP-F* (chromosome segregation in mitosis and telomerase activity), *INHBA* (cell proliferation, metastasis, cellular senescence, and immune evasion), *NEFM* (methylation and immune cell infiltration), and *SAT1* (catabolism of polyamines and ferroptosis). Together, these findings highlight the importance of NFX1-123 in maintaining normal expression patterns that are critical for cancer cell function and behavior and highlight the diverse cellular processes it influences.

Several of these differentially expressed genes highlight how NFX1-123 depletion disrupts essential mechanisms that are relevant to cervical cancer cell survival and virus-associated pathways. For instance, *RBM25* helps to assemble early spliceosomes, driving the splicing of precursor mRNAs, which is vital for cell viability [39,40]. Likewise, proline-rich coiled–coiled 2C protein (PRRC2C) ensures the proper scanning of upstream open reading frames (uORFs) to assure that ribosomes bind to mature ORFs (mORFs) and facilitate translational initiation [41]. Both of these post-transcriptional regulations affect gene expression and play a role in tumor progression when dysregulated [42]. DYNC1H1, a hotspot for HPV16 integration, negatively influences cell viability, proliferation, migration, and invasion when downregulated [43,44,45]. Its dynein light chains, DYNLT1 and DYNLT3, further facilitate HPV 16 infection, [46] underscoring their importance in viral pathogenesis. Ferritin light chain (FTL), which regulates iron storage and is overexpressed in multiple cancers [47,48,49,50], was also observed to be diminished with NFX1-123 depletion. High ferritin levels have been tied to poorer HPV 16 clearance and greater oxidative DNA damage [51].

Additionally, *CENP-F*, which was first identified in HeLa cells [52], was upregulated in cervical cancer and is associated with metastasis [30]. It also has been shown to be a correlative marker with a high expression of telomerase activity in breast cancer [29]. A reduction in CENP-F resulted in cell growth, migration, and invasion inhibition while increasing the ferroptosis, an iron accumulation-mediated cell death mechanism in cervical cancer [38]. Taken together, these findings illustrate how NFX1-123 modulates processes that are central to mRNA processing, cell cycle control, iron metabolism, and viral oncogenesis, highlighting its broad significance in cervical cancer tumor biology.

The three additional genes whose mRNAs were greatly decreased in both scRNAseq and qPCR analyses were *INHBA* (inhibin subunit beta A), *NEFM* (neurofilament medium chain), and *SAT*1 (spermidine/spermine N(1)-acetyltransferase). Although the precise role that these genes may play in cervical cancer remains to be thoroughly investigated, their functional significance has been demonstrated in other cancers and their expression linked to cervical cancer itself. INHBA is a widely expressed transforming growth factor-beta family member, which is a biomarker for some cancers. INHBA is involved in cellular senescence and immune evasion, and it promotes cell proliferation and metastasis in several cancers. In cervical cancer, INHBA overexpression was correlated with pathological features, antitumor immune response, and clinical prognosis [53,54]. NEFM is a component of neurofilaments in neurons [55,56]. Neurofilaments have been implicated in the pathology of cancers, and *NEFM* is located in Ch8p21, where loss of heterozygosity has been shown in several cancers [57,58,59]. Methylation and aberrant expression of neurofilament genes have been detected in renal, hepatocellular, esophageal, and breast cancers, as well as Ewing’s sarcomas [60,61,62,63]. Silencing or low expression of NEFM correlated with breast cancer progression [64], immune cell infiltration, and poor survival [65]. In an intersectional analysis of TCGA methylation-related differentially expressed genes (TCGA-MRDEGs) and GEO-MRDEGs, NEFM was identified as one of the ten overlapping MRDEGs in cervical cancer [66]. SAT1 is a rate-limiting enzyme in the catabolism of polyamines, which regulate translation, replication, and chromatin condensation [67,68,69]. High expression of SAT1 has been demonstrated in glioma, driving poor outcomes [70,71], triple negative breast cancer progression [72], and ferroptosis in endometrial cancers [73].

As we know, NFX1-123 and 16E6 collaborate to regulate hTERT and telomerase activity. The majority of genes that had decreased mRNA levels in CaSki KO cells were associated with RNA binding, transcriptional regulators, and chromatin regulators. We returned to investigate *hTERT* mRNA levels and telomerase activity in cervical cancer cell lines that either had NFX1-123 knocked out or were treated with a small-molecule compound (R428) that decreased the NFX1-123 protein. Additionally, we also tested the synergistic effects of 16E6 and 16E7 knockdown using siRNA. Finally, because CENP-F expression has been associated with telomerase activity in cancers, and we determined that its expression was reduced with NFX1-123 KO, we quantified its protein levels in these cell lines as well. As predicted, telomerase activity and *hTERT* mRNA were reduced with NFX1-123 KO SiHa and CaSki cells, and these were further decreased in combination with 16E6E7 knockdown. So, each protein—NFX1-123 as the host cell protein and 16E6 as the viral oncoprotein—needed to be fully expressed to support hTERT expression and telomerase activity in cervical cancer cell lines. CENP-F protein, like its mRNA, was reduced in NFX1-123 KO CaSki cells (Figure 6D). This points to additional, shared regulation of telomeric DNA maintenance in cervical cancers, beyond the telomerase enzyme and its catalytic subunit hTERT, itself. Telomerase inhibitors have been evaluated for their antitumor effects in both preclinical and clinical settings. Despite the demonstrated preclinical effects of telomerase inhibitors, clinical effectiveness has not been documented with the majority of these compounds [74]. In a randomized phase II clinical trial, the telomerase inhibitor imetelstat was evaluated as a maintenance therapy in advanced non-small-cell lung cancer [75], and this same inhibitor has been studied as a fatty acid metabolism regulator and inducer of ferroptosis in the treatment of acute myeloid leukemia [76]. However, telomerase-targeted therapy is challenging due to the complex regulation of hTERT, the activation of hTERT-independent alternative telomerase (ALT), and the challenging pharmacokinetics of telomerase inhibitors [74]. Our findings suggest that R428, an Axl kinase inhibitor, has the potential to inhibit NFX1-123 and, through NFX1-123, regulate telomerase activity. R428 has been evaluated in preclinical and clinical trials as an anti-cancer compound, yet its effects on telomerase activity have not been evaluated in HPV-associated cancers. Further studies of NFX1-123, its regulation by R428, and their effects on telomerase activity may provide future combination approaches with existing chemotherapy in HPV-associated cancer treatment.

Lastly, we utilized the TCGA database and demonstrated an increase in *CENP-F* and *hTERT* mRNA in HPV-associated cancers beyond the cervix. In HPV-positive head and neck cancers, *CENP-F* and *hTERT* mRNA levels were significantly increased when compared with HPV-negative head and neck cancers (Figure 8). By proxy, this demonstrates an association between NFX1-123 expression, HPV oncogenes including E6, and critical pathways that require virus–host interactions in cancer. Understanding the mechanisms that underpin gene regulation by NFX1-123, and that are dysregulated by HPV in cancers, as well as extending our findings with in vivo validation in a clinical setting, are all critical to study.

## 5. Conclusions

Our findings, identified using scRNAseq of cervical cancer cell lines, objectively demonstrated common RNA, cell cycle, and host–virus interactions that depend on NFX1-123 and newly identified *CENP-F* as a gene whose full expression required high expression of NFX1-123. Further, our results showed the telomerase activity regulation associated with NFX1-123 and HPV16E6 in cervical cancer. Our findings have translational relevance as targeting NFX1-123 to regulate telomerase activity may be a new therapeutic approach to enhance the chemotherapeutic efficacy of the current treatments for HPV-associated cancers. Future research is warranted to better understand NFX1-123 inhibitors and their molecular mechanism of action using in vivo and patient-derived models.

## Data Availability

The original data on single-cell sequencing are available in the SRA database and are publicly available (BioProject ID: PRJNA1257964). The gene expression analysis on *hTERT* and *CENP-F* was performed using TCGA TSVdb (http://www.tsvdb.com/).

## Supplementary Materials

The following supporting information can be downloaded at: https://www.mdpi.com/article/10.3390/cancers17122044/s1, File S1: The original Western blot figures; Table S1: The top 10 downregulated genes in NFX1-123 KO CaSki cells compared with CTL and their functions.

## Abbreviations

The following abbreviations are used in this manuscript:

NFX1-123	Nuclear transcription factor, X-box binding 1-123
hTERT	Human telomerase reverse transcriptase
scRNAseq	Single-cell RNA sequencing
CENP-F	Centromere protein F
