# Supplementary material for: Reduction of NFX1-123 and HPV 16 E6 and E7 Decreased Telomerase and CENP-F in Cervical Cancer Cell Lines
Source: Cancers (Basel). 2025 Jun 19;17(12):2044. doi: 10.3390/cancers17122044 (PMC12190452; doi:10.3390/cancers17122044)
Supplement: Supplementary file 1 [file cancers-17-02044-s001.zip › cancers-3681275-original Western blot figures-File S1.pdf]

Figure 1A

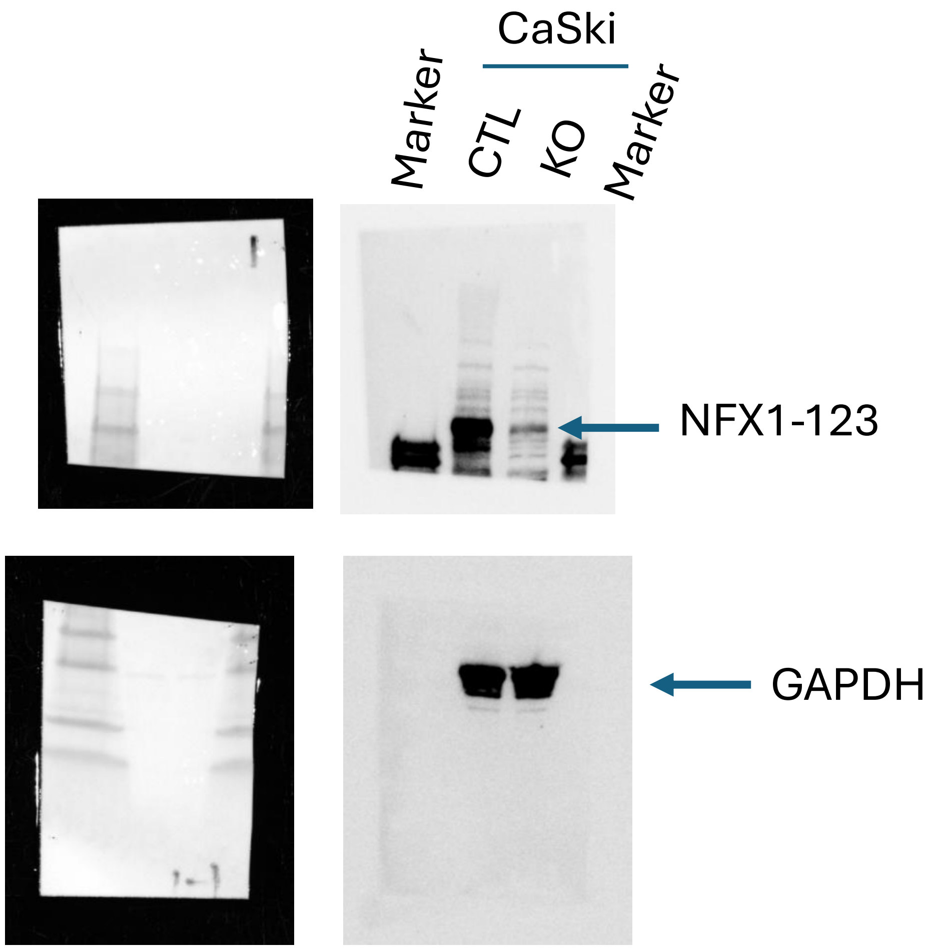

Figure 6A

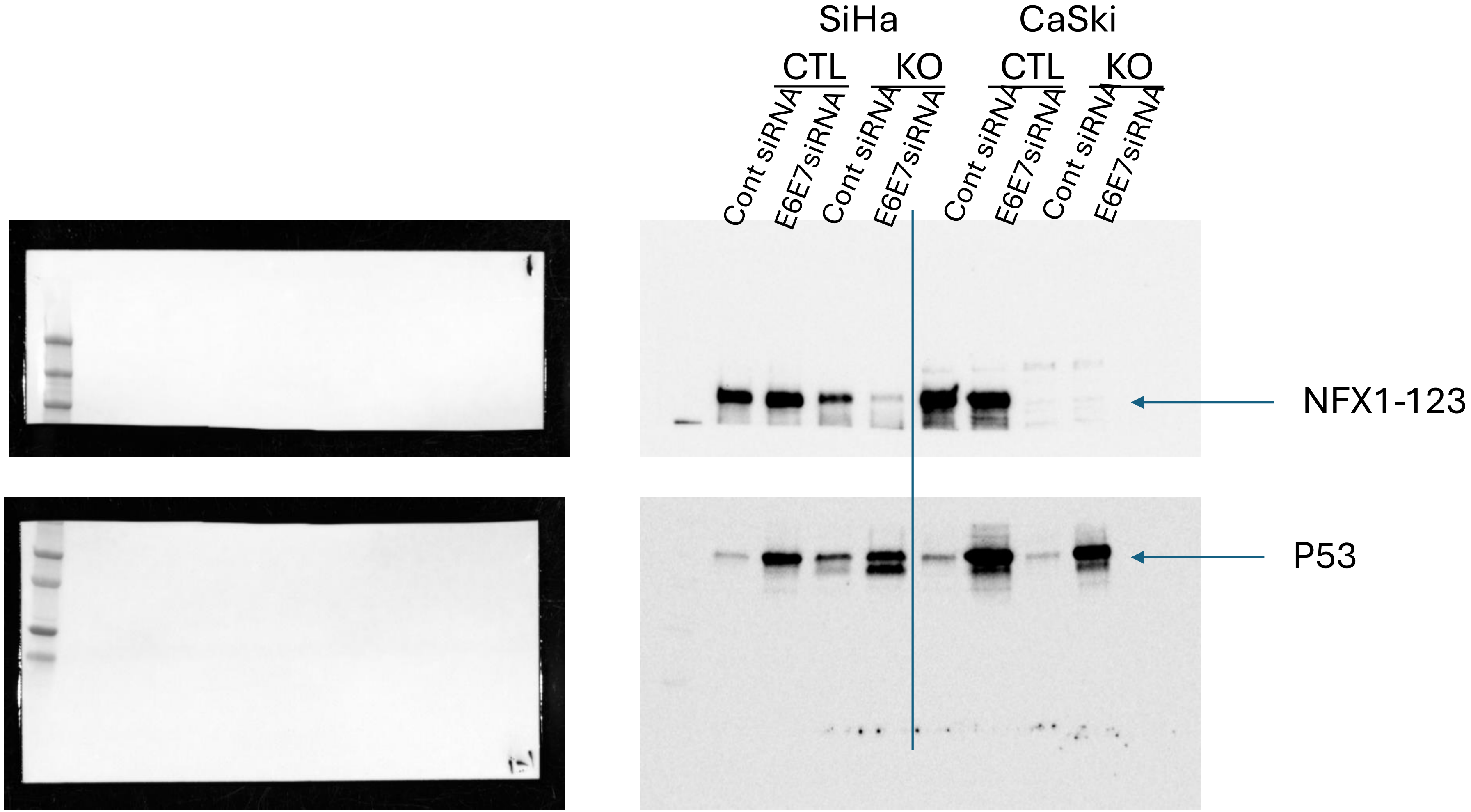

SiHa
CaSki
CTL
KO
CTL
KO
Cont siRNA
E6E7siRNA
Cont siRNA
E6E7siRNA
Cont siRNA
E6E7siRNA
Cont siRNA
E6E7siRNA
NFX1-123
P53

Figure 6A

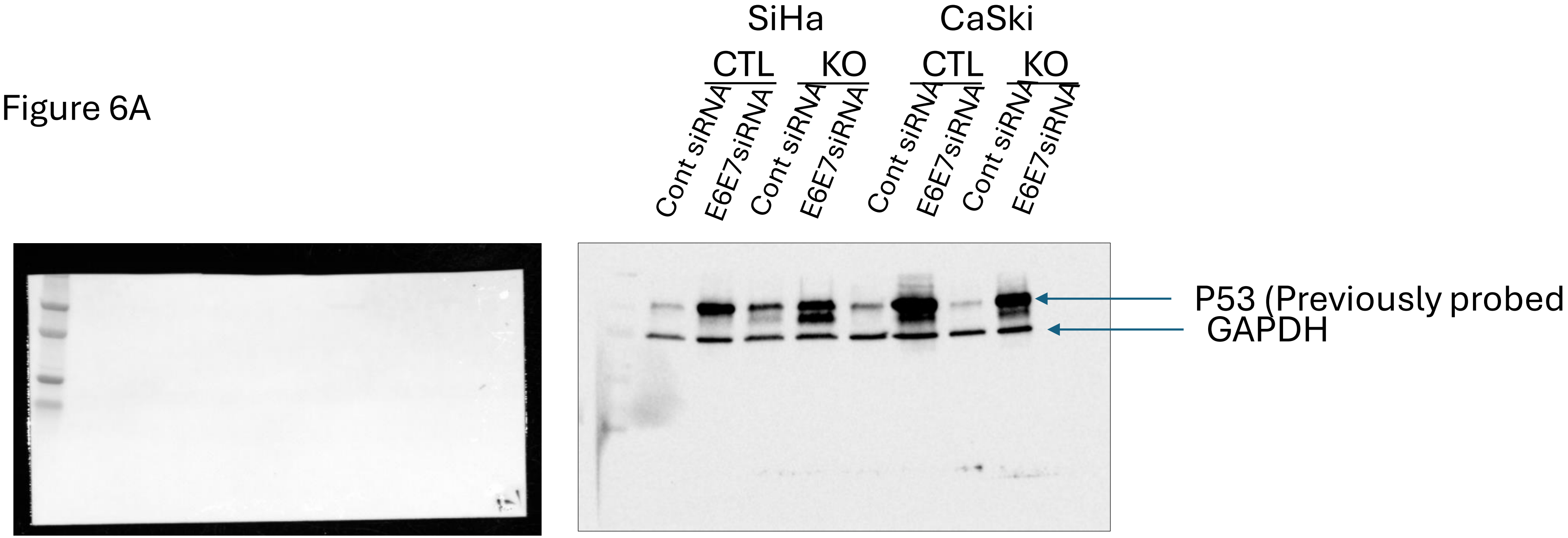

SiHa
CaSki
CTL
KO
CTL
KO
Cont siRNA
E6E7siRNA
Cont siRNA
E6E7siRNA
Cont siRNA
E6E7siRNA
Cont siRNA
E6E7siRNA
P53 (Previously probed
GAPDH

Figure 6B (Left panel)

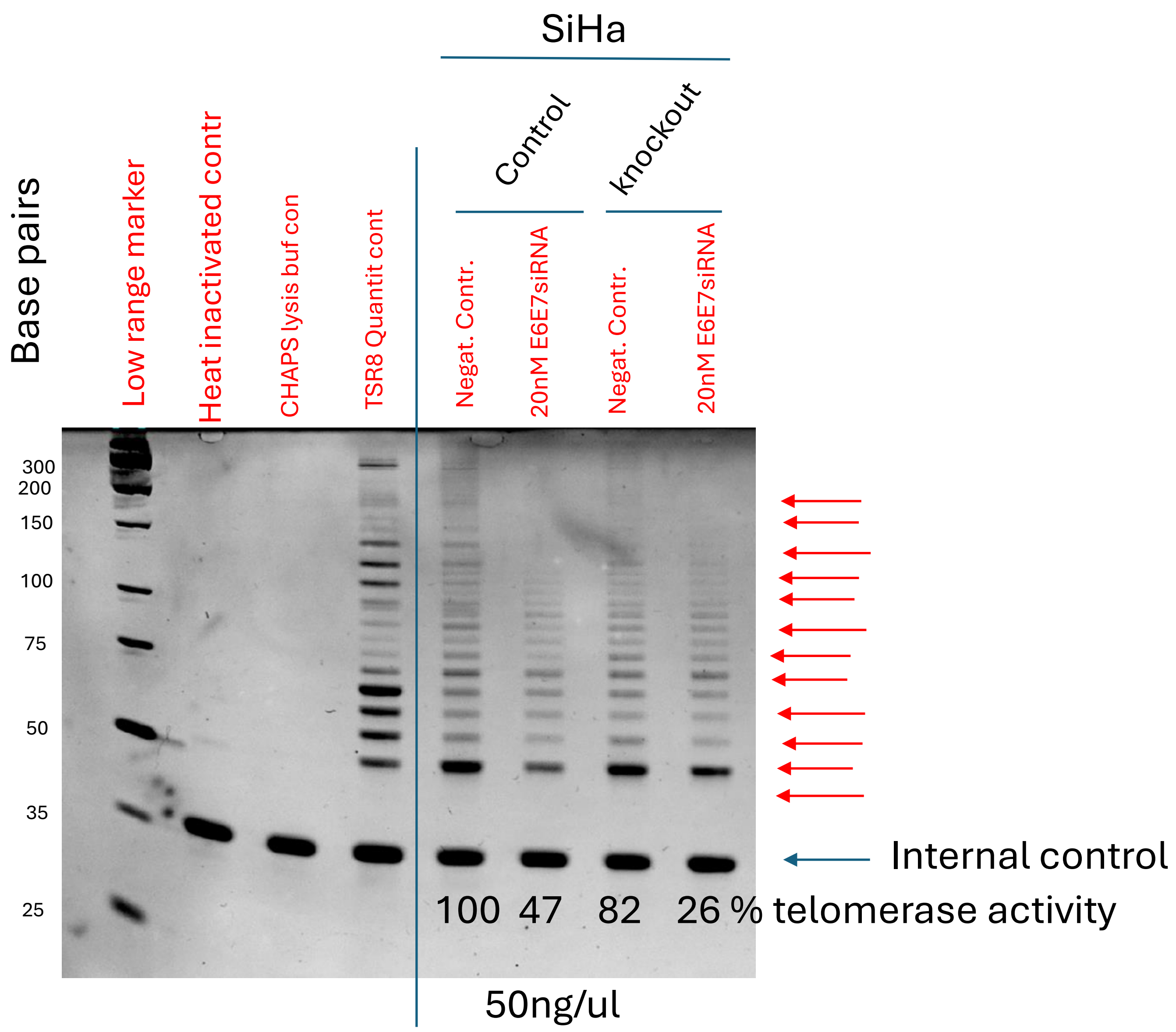

Figure 6B (Right panel)

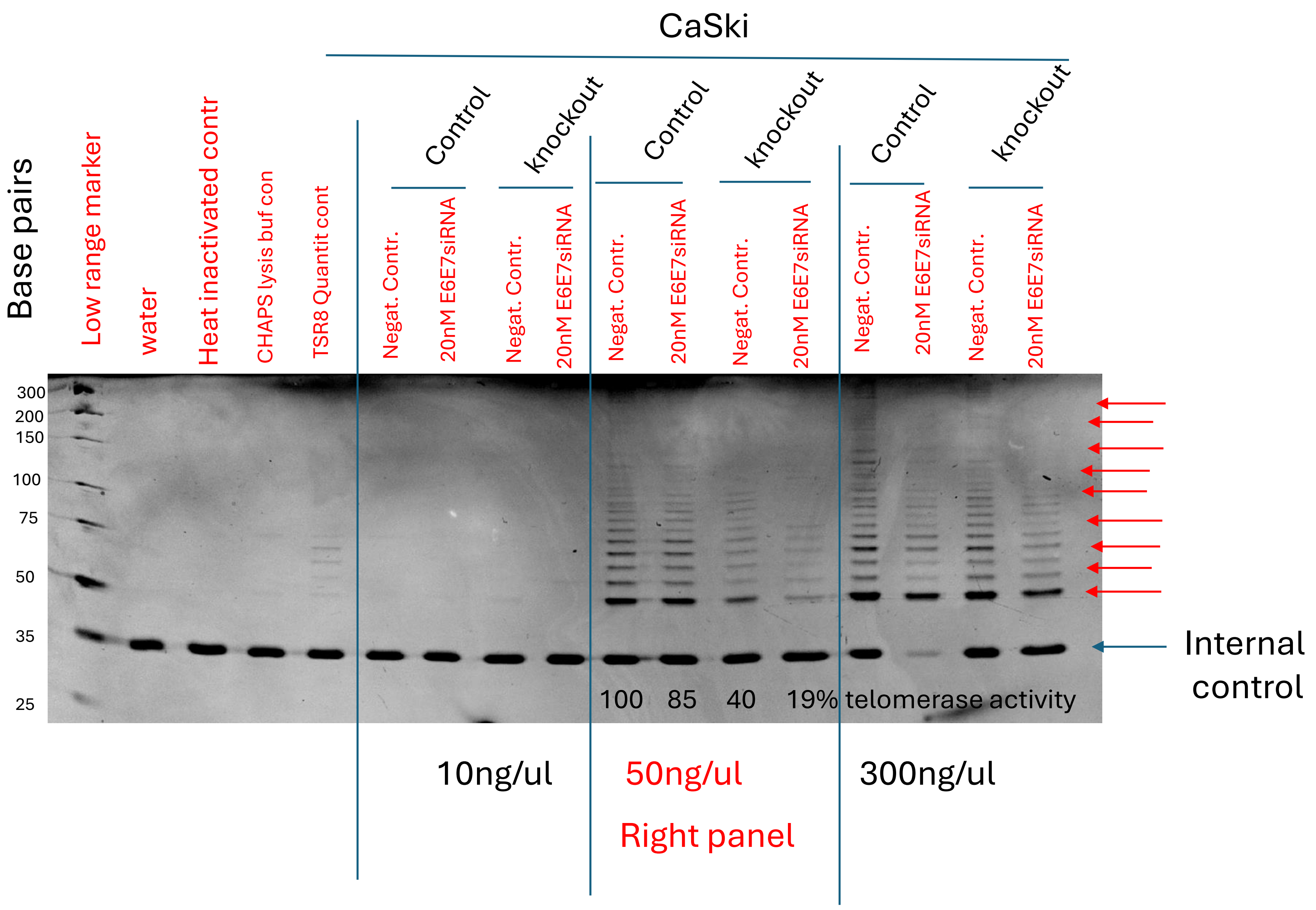

Figure 6D

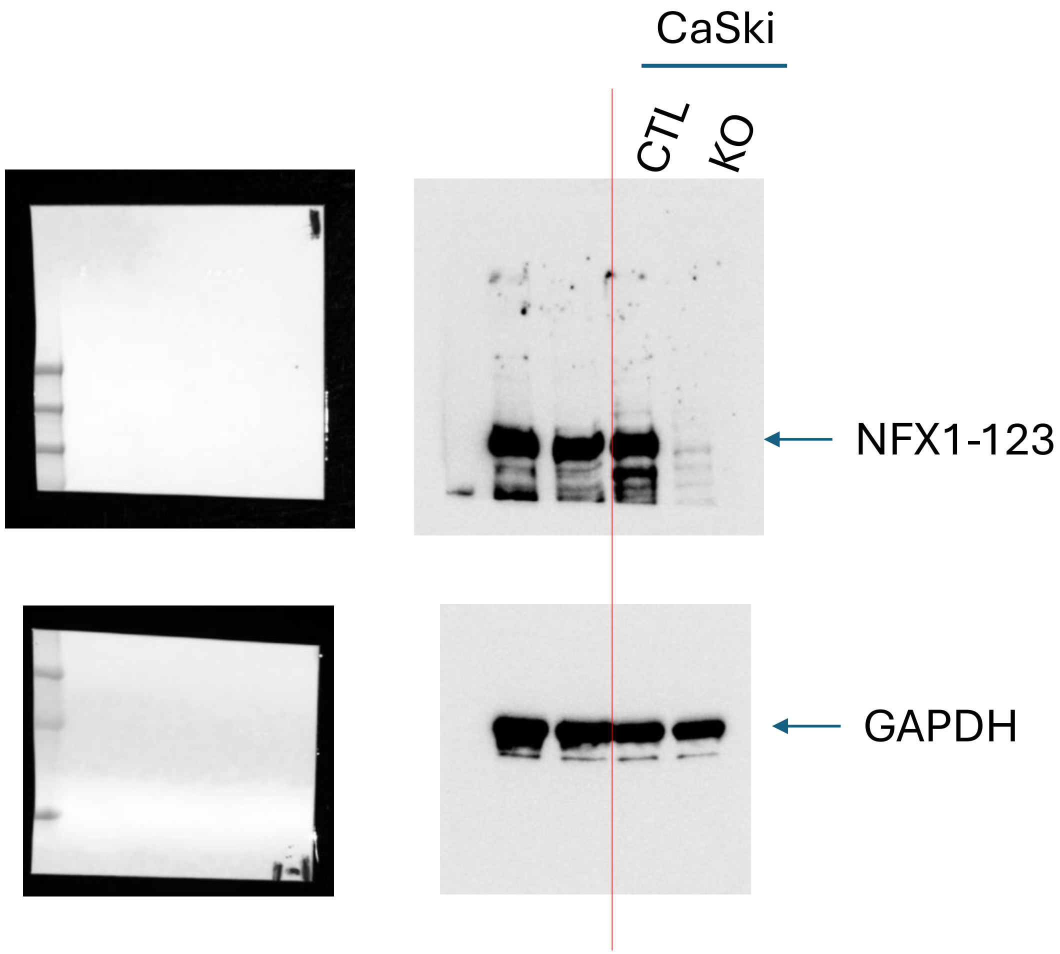

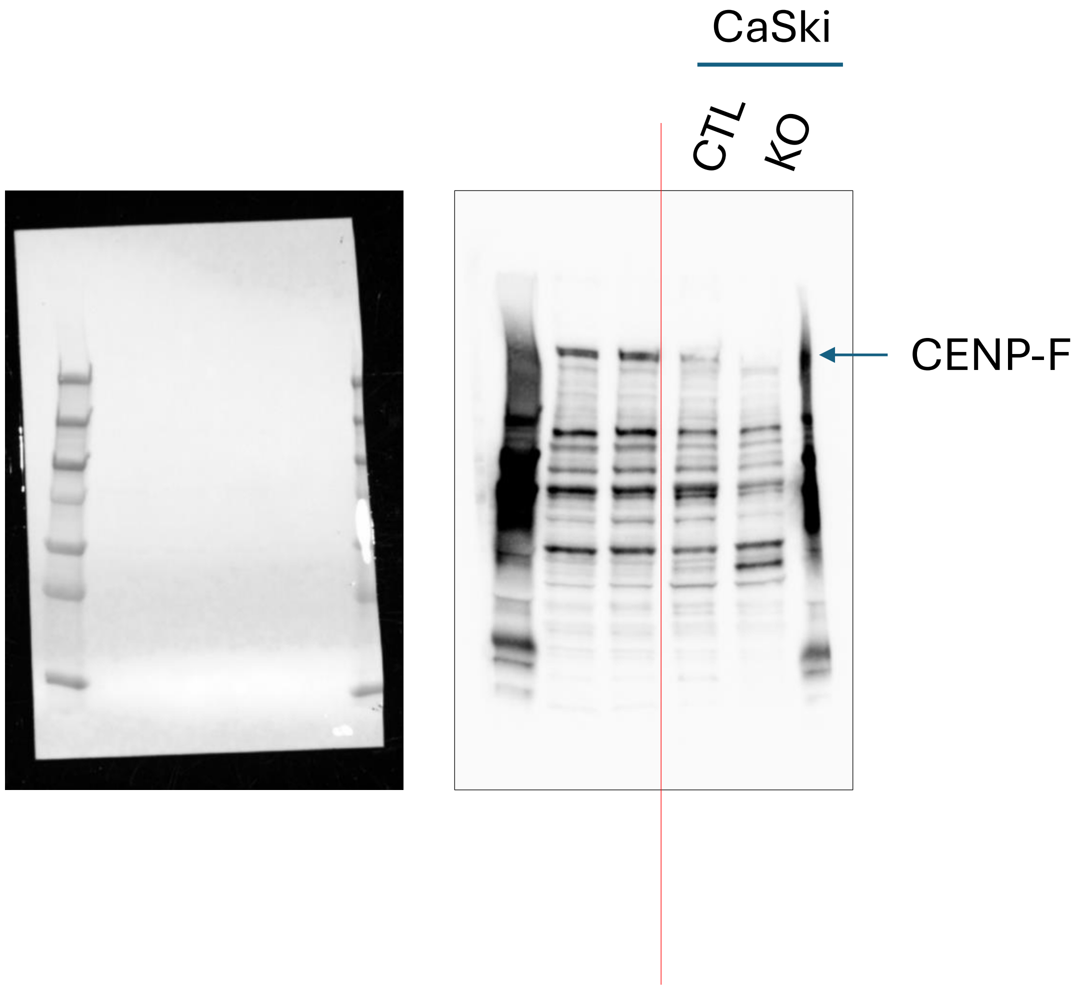

Figure 6D

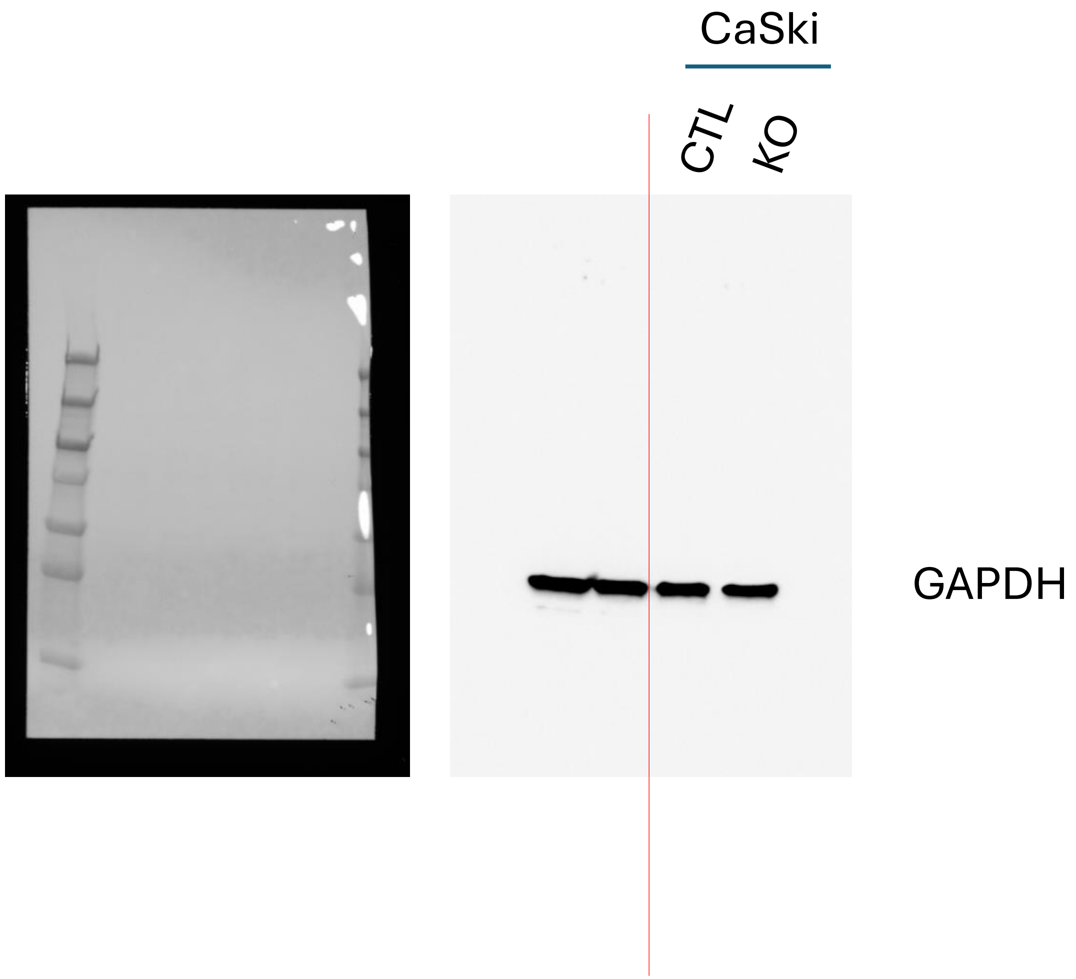

Figure 7A

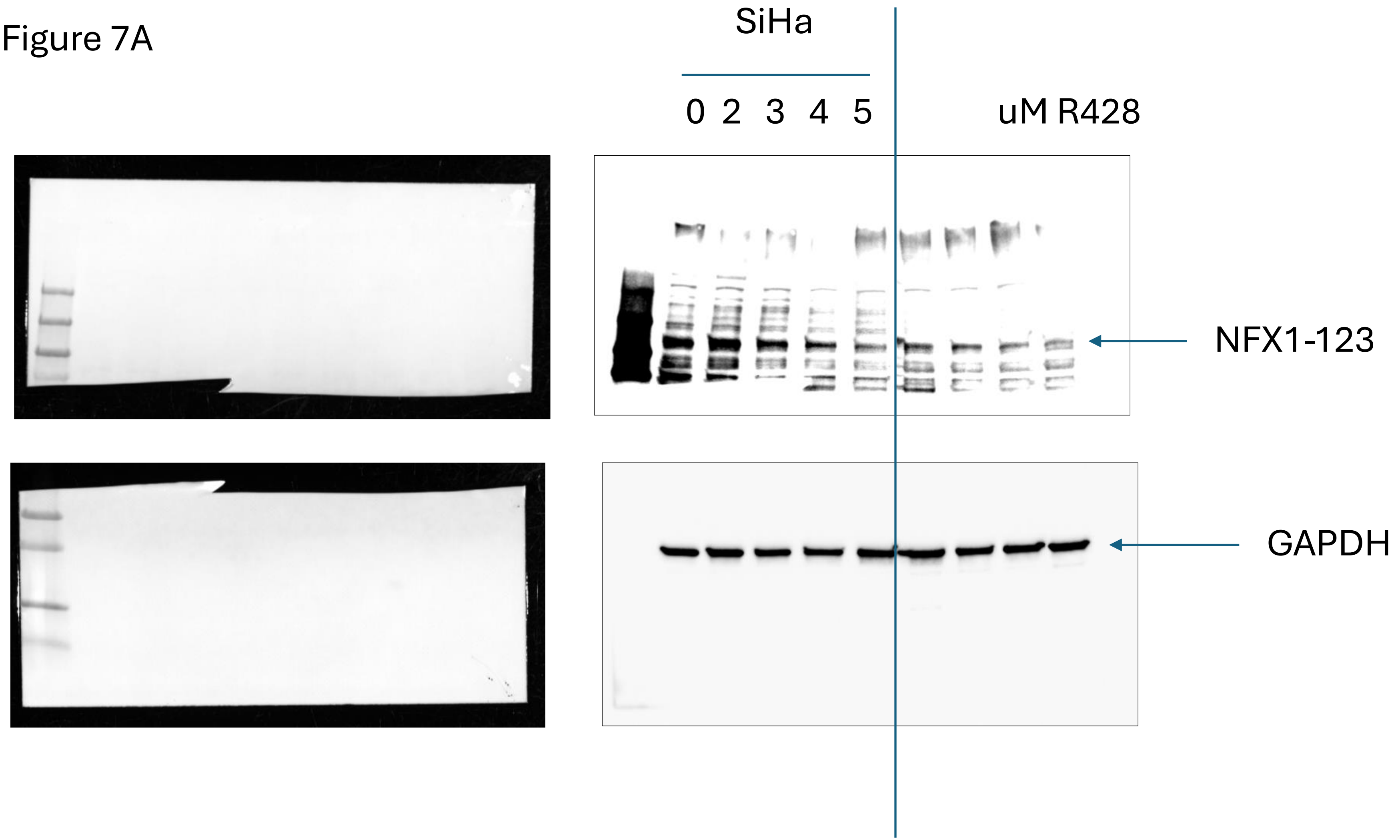

Figure 7A

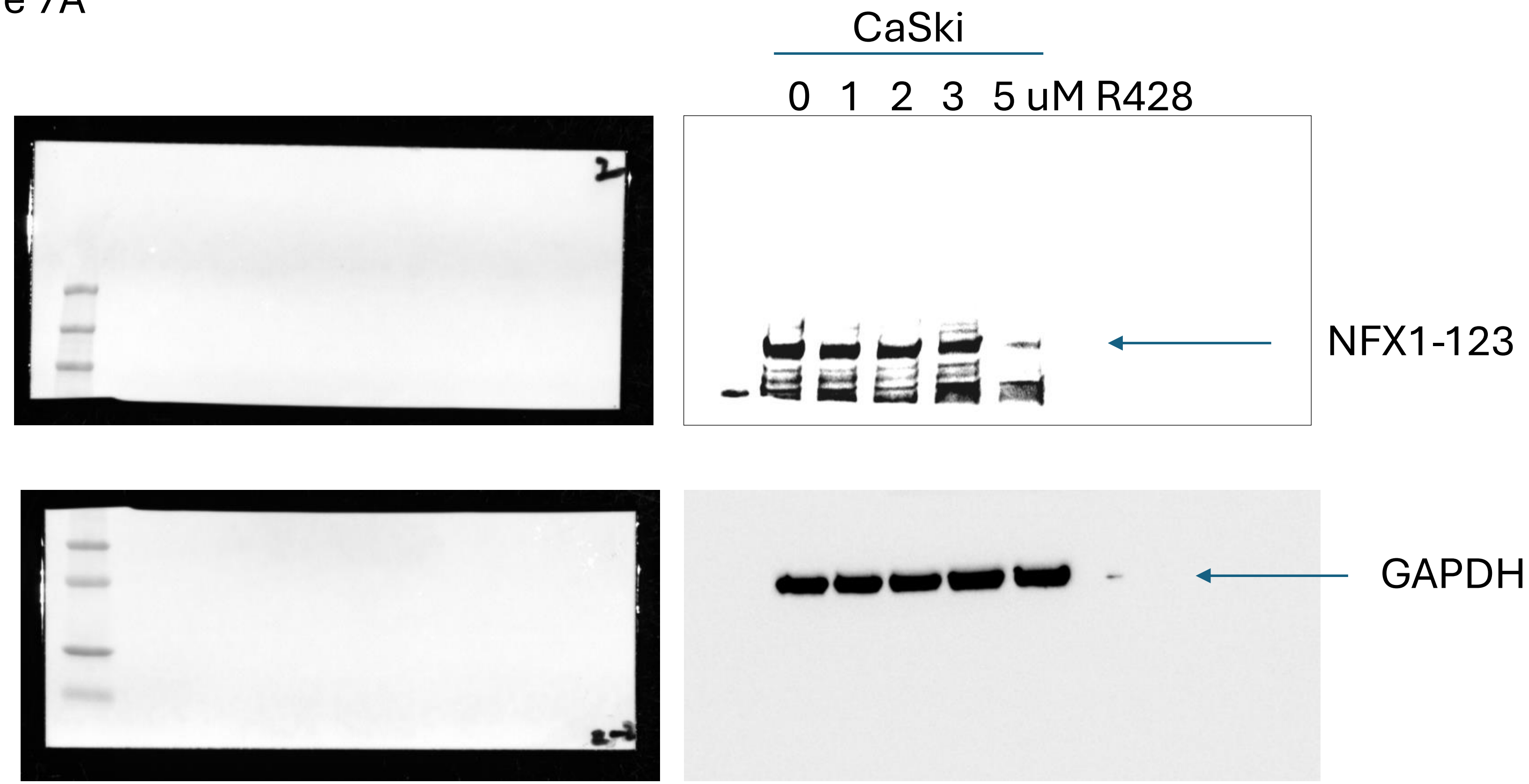

Figure 7B

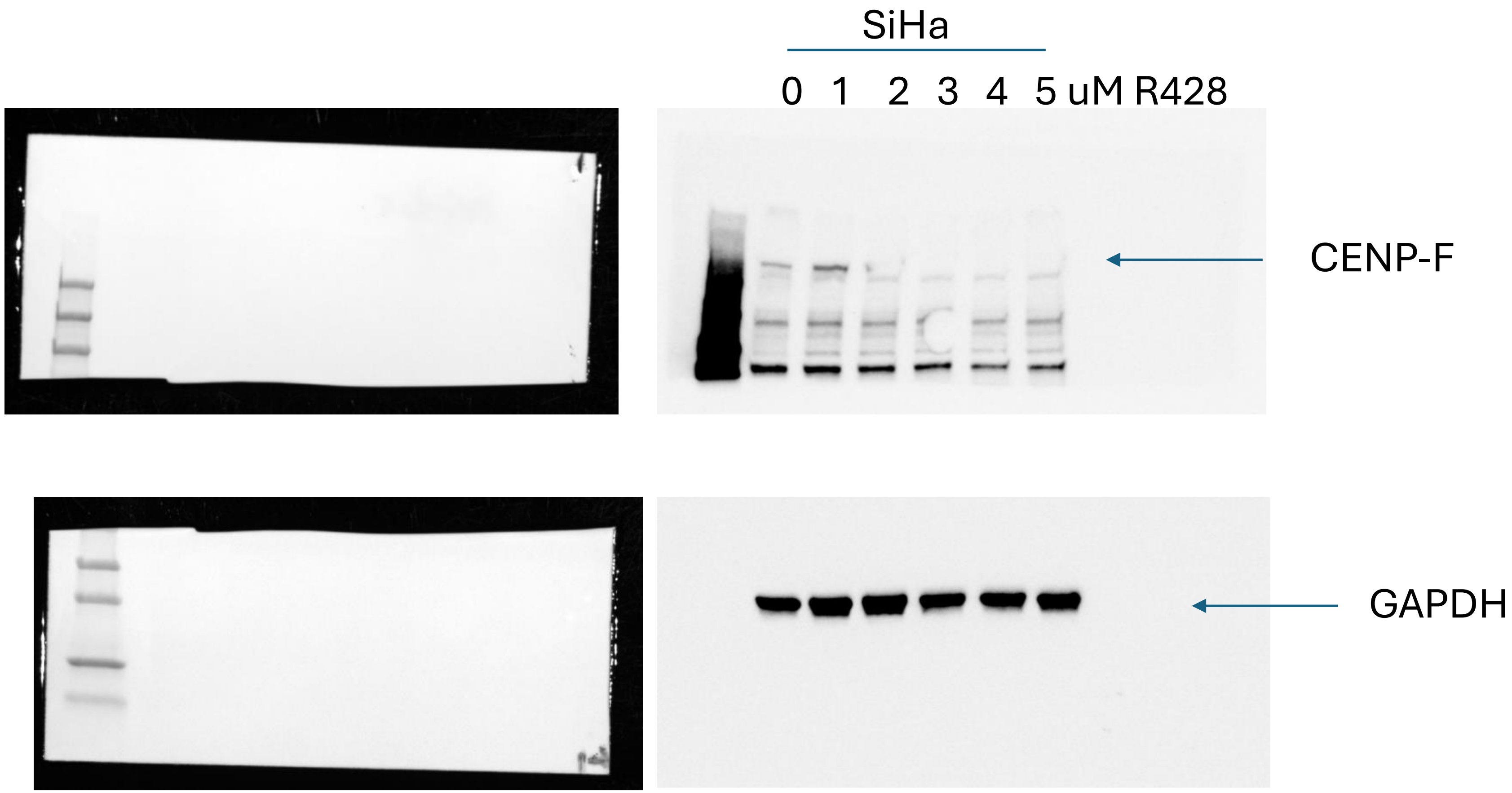

Figure 7B

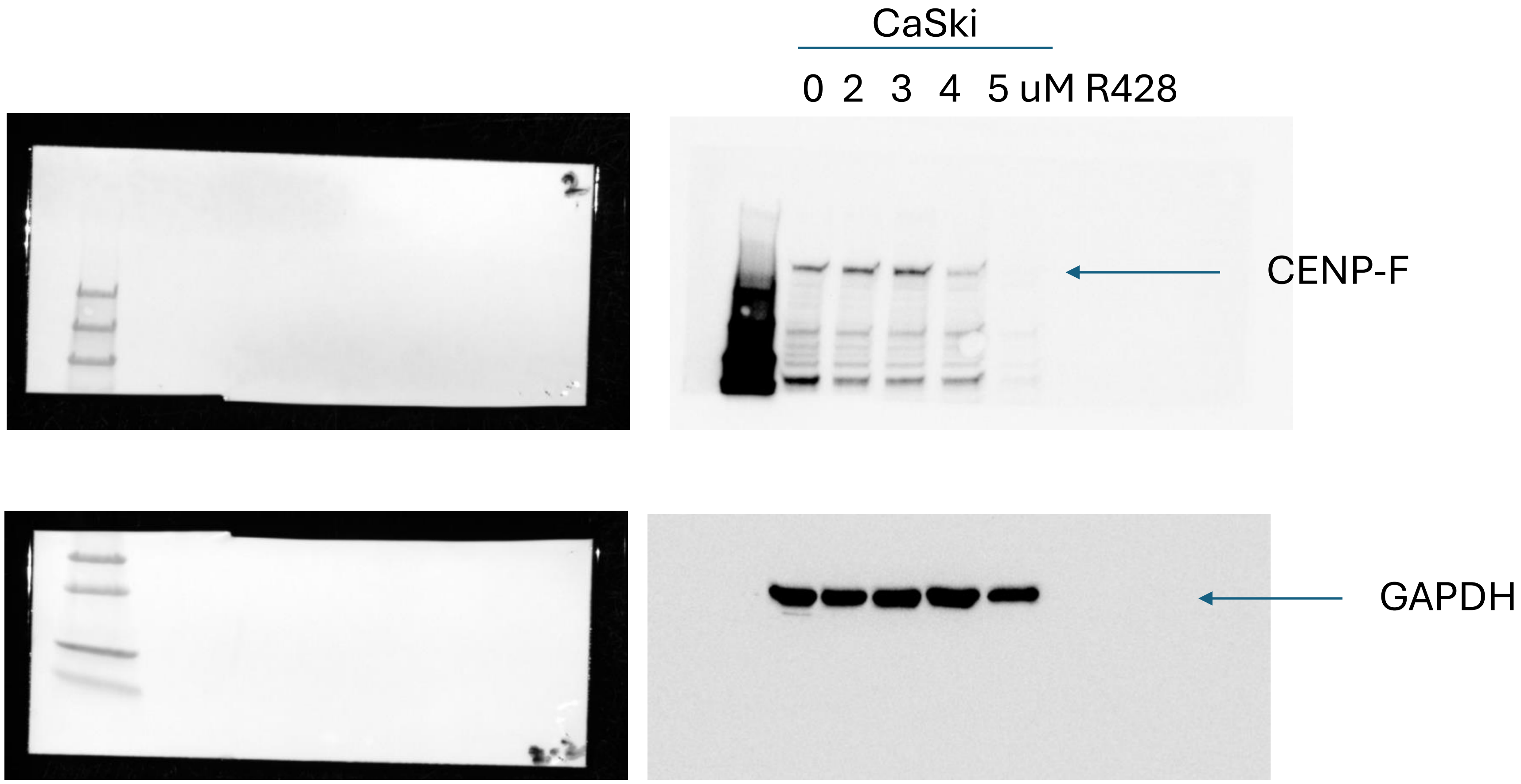

Figure 7E

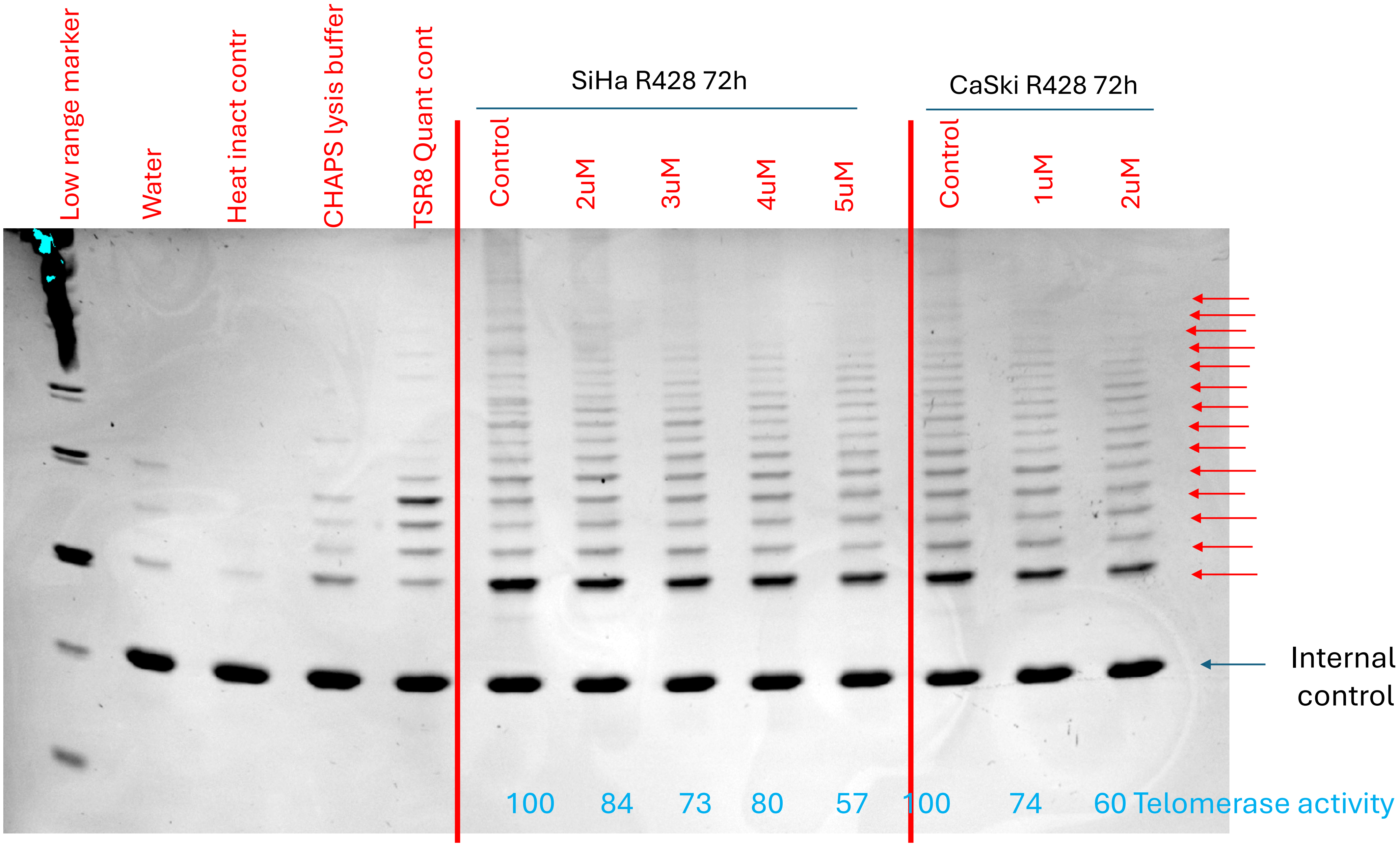
Low range marker
Water
Heat inact contr
CHAPS lysis buffer
TSR8 Quant cont
SiHa R428 72h
Control
2uM
3uM
4uM
5uM
CaSki R428 72h
Control
1uM
2uM
Internal control
100
84
73
80
57
100
74
60 Telomerase activity
